# Target inhibition of galectin-3 by inhaled TD139 in patients with idiopathic pulmonary fibrosis

**DOI:** 10.1183/13993003.02559-2020

**Published:** 2021-05-27

**Authors:** Nikhil Hirani, Alison C. MacKinnon, Lisa Nicol, Paul Ford, Hans Schambye, Anders Pedersen, Ulf J. Nilsson, Hakon Leffler, Tariq Sethi, Susan Tantawi, Lise Gravelle, Robert J. Slack, Ross Mills, Utsa Karmakar, Duncan Humphries, Fredrik Zetterberg, Lucy Keeling, Lyn Paul, Philip L. Molyneaux, Feng Li, Wendy Funston, Ian A. Forrest, A. John Simpson, Michael A. Gibbons, Toby M. Maher

**Affiliations:** 1 MRC Centre for Inflammation Research, The Queen's Medical Research Institute, University of Edinburgh, Edinburgh, UK; 2 Galecto, Copenhagen, Denmark; 3 Dept of Chemistry, Lund University, Lund, Sweden; 4 Dept of Laboratory Medicine, Lund University, Lund, Sweden; 5 Exploristics, Belfast, UK; 6 National Institute for Health Research Respiratory Clinical Research Facility, Royal Brompton and Harefield NHS Foundation Trust, and Fibrosis Research Group, National Heart and Lung Institute, Imperial College London, London, UK; 7 Translational and Clinical Research Institute, Newcastle University, Newcastle upon Tyne, UK; 8 Respiratory Medicine Unit, Royal Victoria Infirmary, Newcastle upon Tyne Hospitals NHS Foundation Trust, Newcastle upon Tyne, UK; 9 Respiratory Dept, Institute of Biomedical and Clinical Science, Royal Devon and Exeter NHS Foundation Trust, Medical School, University of Exeter, Exeter, UK; 10 Keck School of Medicine, University of Southern California, Los Angeles, CA, USA

## Abstract

Galectin (Gal)-3 is a profibrotic β-galactoside-binding lectin that plays a key role in the pathogenesis of idiopathic pulmonary fibrosis (IPF) and IPF exacerbations. TD139 is a novel and potent small-molecule inhibitor of Gal-3.

A randomised, double-blind, multicentre, placebo-controlled, phase 1/2a study was conducted to assess the safety, tolerability, pharmacokinetics and pharmacodynamics of inhaled TD139 in 36 healthy subjects and 24 patients with IPF. Six dose cohorts of six healthy subjects were evaluated (4:2 TD139:placebo ratio) with single doses of TD139 (0.15–50 mg) and three dose cohorts of eight patients with IPF (5:3 TD139:placebo ratio) with once-daily doses of TD139 (0.3–10 mg) for 14 days.

Inhaled TD139 was well tolerated with no significant treatment-related side-effects. TD139 was rapidly absorbed, with mean time taken to reach maximum plasma concentration (*C*_max_) values ranging from 0.6 to 3 h and a plasma half-life (*T*_1/2_) of 8 h. The concentration of TD139 in the lung was >567-fold higher than in the blood, with systemic exposure predicting exposure in the target compartment. Gal-3 expression on alveolar macrophages was reduced in the 3 and 10 mg dose groups compared with placebo, with a concentration-dependent inhibition demonstrated. Inhibition of Gal-3 expression in the lung was associated with reductions in plasma biomarkers centrally relevant to IPF pathobiology (platelet-derived growth factor-BB, plasminogen activator inhibitor-1, Gal-3, CCL18 and YKL-40).

TD139 is safe and well tolerated in healthy subjects and IPF patients. It was shown to suppress Gal-3 expression on bronchoalveolar lavage macrophages and, in a concerted fashion, decrease plasma biomarkers associated with IPF progression.

## Abstract 2

**TD139 is a potent inhibitor of galectin-3, a key driver of fibrosis in the lung. In this phase 1/2a clinical study, inhaled TD139 was safe, well tolerated, and demonstrated target engagement and decreased plasma biomarkers associated with IPF progression.** https://bit.ly/2JREKx6

## Introduction

Idiopathic pulmonary fibrosis (IPF) is a progressive, irreversible, ultimately fatal lung disease characterised by a progressive decline in lung function. The median survival is 3 years and incidence is increasing. The underlying pathogenesis of IPF is unknown; however, it most likely arises as a result of repeated alveolar epithelial cell injury and subsequent aberrant healing. It is considered likely that multiple intrinsic and environmental triggers might lead to IPF [1, 2]. Two antifibrotic therapies have gained regulatory approval for use in IPF: pirfenidone, which has an uncertain mechanism of action, and nintedanib, which is a mixed tyrosine kinase inhibitor [3, 4]. Both drugs have moderate efficacy, but 20–30% of patients discontinue treatment or have dose-limiting side-effects. Thus, safe, effective treatments for IPF are urgently required. Numerous early-phase clinical trials have been performed in IPF, but few of these have progressed to phase 2b or 3 trials. One of the reasons for this attrition from early- to late-phase trials is the lack of confidence that the study drug engages with its target in the human disease state. This issue is not unique to IPF.

Galectin (Gal)-3 is a central regulator of fibrosis in the lung with expression upregulated in bronchoalveolar lavage (BAL) fluid and serum of IPF patients, and further elevation observed during exacerbations [5, 6]. The profibrotic function of Gal-3 is multifactorial due to its ability to cross-link and promote signalling *via* multiple cell surface receptors including integrins and growth factor receptors [7], such as transforming growth factor-β [6, 8], vascular endothelial growth factor [9] and platelet-derived growth factor (PDGF) receptors [10, 11]. Constitutive global deficiency of Gal-3 leads to attenuated fibrosis in murine models [6, 12–14].

TD139 is a 3,3′-bis-(4-aryltriazol-1-yl) thio-digalactoside Gal-3 inhibitor with high affinity for the Gal-3 carbohydrate recognition domain that has shown efficacy in murine models of lung fibrosis [6, 15]. The antifibrotic potential of TD139 centres around the inhibition of the recruitment and expansion of Gal-3-secreting macrophages that drive local myofibroblast activation [14, 16, 17]. TD139 has been shown pre-clinically to exhibit effects on all of the key IPF cell types: modulating macrophage phenotype/Gal-3 expression and fibroblast activation, reducing the effects of key profibrotic growth factors that act on myofibroblasts, and inhibiting epithelial–mesenchymal transition [6, 15, 16, 18, 19].

The aim of this study was to investigate the safety, pharmacokinetics (PK) and pharmacodynamics (PD) profile of TD139 when administered *via* a dry powder inhaler in healthy subjects and IPF patients.

## Materials and methods

### Study design

This study was a randomised, double-blind, multicentre, placebo-controlled, dose-escalation study investigating the safety, tolerability, PK and PD properties of TD139, a Gal-3 inhibitor administered by inhalation, in healthy subjects (part 1) and patients with IPF (part 2). The study is registered at ClinicalTrials.gov with identifier number NCT02257177. Part 1 was completed at Simbec Research (Merthyr Tydfil, UK) and part 2 was undertaken at four hospitals in the UK (Edinburgh Royal Infirmary, Edinburgh; Royal Brompton Hospital, London; Royal Victoria Infirmary, Newcastle upon Tyne; and the Royal Devon and Exeter NHS Trust, Exeter). Independent ethics committee approval was obtained prior to initiation of the study. Screening in study centres was performed within 28 days prior to randomisation and first dosing (day 1). The trial was registered prospectively as a two-part phase 1 study: part 1 in healthy volunteers commenced in September 2014 and part 2 in patients with IPF commenced in March 2015.

In part 1 of the study, following an initial screening process, subjects were randomly assigned in a 4:2 ratio to receive a single dose of TD139 (0.15, 1.5, 3, 10, 20 or 50 mg) or placebo *via* a dry powder inhaler (Plastiape/Berry Bramlage, Lohne, Germany) (randomisation code generated using the PROC PLAN procedure of SAS). All subjects, site and study sponsor personnel were blinded to the study group assignments throughout the study. TD139 or placebo was administered in the morning of day 1 fasted (after an overnight fast of at least 10 h). Subjects were discharged 24 h post-dose (day 2) provided there were no safety concerns, and returned for three outpatient visits on days 3, 8 and 14 post-dose, with a post-study follow-up between days 26 and 30. Safety and PK assessments were made at predetermined time-points.

In part 2 of the study, following an initial screening process, patients with a multidisciplinary team (MDT)-confirmed diagnosis of definite or probable IPF according to current international consensus criteria [1] were randomly assigned in a 5:3 ratio to receive a dose of TD139 (0.3, 3 or 10 mg) or placebo *via* a dry powder inhaler (Plastiape/Berry Bramlage) once daily for 14 days (randomisation code generated using the PROC PLAN procedure of SAS). All patients, investigators, sites and study sponsor personnel were blinded to the study group assignments throughout the study. Patients underwent study-specific assessments on days 2, 3, 7, 14 and 15, and a post-study assessment was carried out 26–30 days after the last day of the study period. Assessments included vital signs (supine blood pressure, pulse, oxygen saturation, oral temperature and respiratory rate), spirometry, 12-lead ECG, physical examination, laboratory safety screen (haematology, biochemistry, urinalysis and urinary drugs of abuse screen) and blood sampling for PK measurements. All adverse events and concomitant medications were recorded from the time of the screening visit until the post-study follow-up visit. BAL was performed at day −1 (24 h before the first dose) and at day 14 (2 h after the last dose). The BAL procedure was performed as per international guidelines [20]. Briefly, 240 mL of 0.9% saline at room temperature was instilled in 40 mL aliquots into the right middle lobe and aspirated by suction after each aliquot.

**FIGURE 1:**
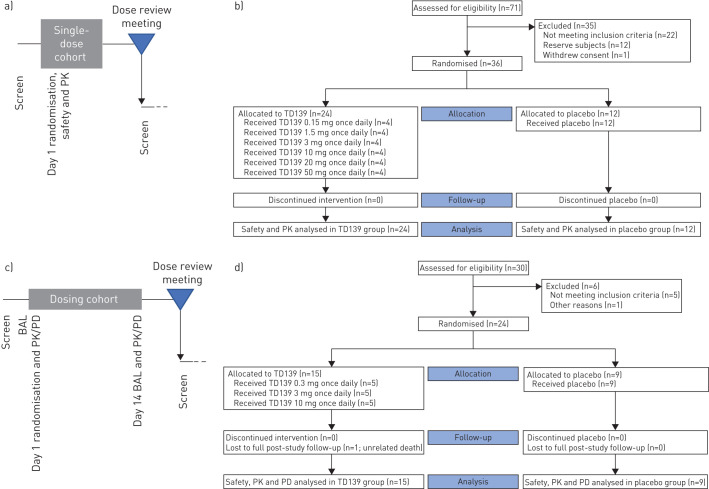
Schematic of the study design and CONSORT (Consolidated Standards of Reporting Trials) diagrams. PK: pharmacokinetics; BAL: bronchoalveolar lavage; PD: pharmacodynamics. a) Part 1 (healthy subjects). Each single-dose cohort consisted of six healthy subjects randomised 4:2 to active and placebo. A minimum of five patients’ data was required in each cohort prior to data review. b) CONSORT diagram for part 1. c) Part 2 (idiopathic pulmonary fibrosis patients). Each repeat-dose cohort consisted of eight subjects randomised 5:3 to active and placebo. A minimum of seven patients’ data was required in each cohort prior to data review. d) CONSORT diagram for part 2.

Following completion of dosing for the final subject in each dosing cohort, an interim dose review meeting was held with a safety committee consisting of the principal investigator, medical monitor, pharmacokineticist and statistician to review the safety, tolerability and PK data (study design schematic shown in figure 1). The study statistician and pharmacokineticist were unblinded at the end of each cohort in order to conduct the analyses required for dose review decisions. The primary end-point was the safety and tolerability of single or multiple doses of TD139 in healthy subjects or IPF patients, respectively. Secondary end-points included evaluation of the PK properties of TD139, expression of Gal-3 in lung and blood, and changes in lung macrophage phenotype and plasma biomarkers following treatment.

### Study participants

The healthy subject population was comprised of 36 male subjects aged 23–53 years. The patient population was comprised of 24 patients aged 45–85 years with a consensus MDT diagnosis of IPF based on the American Thoracic Society/European Respiratory Society/Japanese Respiratory Society/Latin American Thoracic Association criteria [1]. Subjects had forced vital capacity (FVC) ≥45% predicted, forced expiratory volume in 1 s (FEV_1_)/FVC ≥0.7, oxygen saturation >90% on air, diffusing capacity of the lung for carbon monoxide >25% predicted, and were judged by investigators to be safely able to undergo BAL prior to drug treatment and again after 14 days of treatment. Patients receiving oral corticosteroids or any approved or investigational antifibrotic therapies for IPF within 4 weeks of initial screening were excluded from the study. The baseline demographics are shown in tables 1 and 2, and a full list of inclusion and exclusion criteria is presented in supplementary table S1.

**TABLE 1:** Baseline demographics: part 1 (healthy subjects)

	**TD139**						**Placebo**	**Total**
	**0.15 mg**	**1.5 mg**	**3 mg**	**10 mg**	**20 mg**	**50 mg**		
**Subjects**	4	4	4	4	4	4	12	36
**Age years**	42.3±9.0	29.8±6.8	39.3±10.2	29.8±2.1	30.8±9.5	32.8±9.9	35.6±7.5	34.6±8.4
**BMI kg·m^−2^**	25.6±5.1	30.3±2.7	25.9±3.5	25.1±2.7	23.8±1.2	25.7±1.4	25.6±2.4	25.9±3.1
**Height m**	1.84±0.12	1.75±0.10	1.77±0.07	1.75±0.03	1.73±0.05	1.85±0.11	1.79±0.07	1.78±0.08
**Weight kg**	86.0±15.5	93.1±12.8	80.8±9.2	76.0±5.9	71.4±1.5	88.2±13.4	81.8±8.8	82.3±11.0

Data are presented as n or mean±sd. BMI: body mass index. All subjects in part 1 were male.

**TABLE 2:** Baseline demographics: part 2 (idiopathic pulmonary fibrosis patients)

	**TD139**			**Placebo**	**Total**
	**0.3 mg**	**3 mg**	**10 mg**		
**Subjects**	5	5	5	9	24
**Age years**	69.0±6.3	73.6±5.9	79.2±2.8	72.9±4.6	73.5±5.8
**Sex**					
Male	4 (80.0)	5 (100.0)	5 (100.0)	9 (100.0)	23 (95.8)
Female	1 (20.0)	0 (0)	0 (0)	0 (0)	1 (4.2)
**BMI kg·m^−2^**	28.8±1.7	25.0±1.4	26.3±2.2	28.4±2.4	27.3±2.4
**Height m**	1.73±0.06	1.77±0.05	1.74±0.09	1.78±0.05	1.76±0.06
**Weight kg**	86.3±3.9	78.5±5.7	80.1±10.6	90.2±7.4	84.8±8.5
**FVC % pred**	108.0±19.6	81.8±16.0	98.0±13.1	91.7±14.0	94.3±16.8
**FEV_1_ % pred**	104.0±19.6	88.4±16.8	106.4±17.7	94.9±13.6	97.9±16.8

Data are presented as n, mean±sd or n (%). BMI: body mass index; FVC: forced vital capacity; FEV_1_: forced expiratory volume in 1 s.

### Study drug

TD139 was administered once daily *via* a dry powder inhaler. For part 2, a dose range between 0.3 and 10 mg once daily was predicted to provide pharmacologically relevant exposures based on *in vitro* and *in vivo* pharmacology studies.

### Pharmacokinetics

Full plasma PK profiles were conducted on days 1 (first day of dosing) and 14 (last day of dosing). Plasma, BAL alveolar macrophage and BAL fluid concentrations of TD139 were analysed using a validated analytical method based on protein precipitation, followed by high-performance liquid chromatography/mass spectrometry analysis. Standard noncompartmental methods were applied to derive PK parameters using Phoenix WinNonlin version 6.3 (Certara, Sheffield, UK). Reported parameters were maximum plasma concentration (*C*_max_), minimal plasma concentration (24 h post-dose) (*C*_min_), plasma half-life (*T*_1/2_) and area under the concentration time curve from time of dosing extrapolated to infinity (AUC_0−inf_). Drug compartment measurements in BAL alveolar macrophages and fluid were determined as described previously [21]. Epithelial lining fluid (ELF) concentrations of TD139 were calculated as described previously with urea measured using a QuantiChrom urea assay kit (BioAssay Systems, CA, USA) [22].

### Pharmacodynamics end-points

BAL cells were separated from fluid by centrifugation, and cells were collected, stained with antibodies (supplementary material) and then fixed before analysis using a LSR Fortessa flow cytometer (Becton Dickinson, Franklin Lakes, NJ, USA). Data were analysed using FlowJo software (Tree Star, Ashland, OR, USA). Macrophages were identified as having high side scatter properties and human leukocyte antigen-DR positivity. Gal-3 expression (mean fluorescence intensity) was determined from the macrophage gate (supplementary figures S1 and S2). A range of pre-selected plasma biomarkers known to be involved in Gal-3 pathways and/or suggested to be putative biomarkers in IPF were also investigated (supplementary material and supplementary table S2). Samples were analysed by magnetic Luminex assay (R&D Systems, Minneapolis, MN, USA) or ELISA.

### Statistical analysis

For part 2 of the study, placebos were combined from the three dose-specific cohorts into a pooled control group (n=9). For alveolar macrophage Gal-3 expression and the plasma biomarkers, ANCOVA was performed for percentage of baseline ((day 14/day 1)×100). The ANCOVA model included effects for treatment group and baseline (day 1) value. The least square means for each treatment group, the difference in least square means for each active dose compared with control with associated 95% two-sided confidence interval as well as the p-value for treatment difference were obtained. As some imbalance was seen in demographic and baseline characteristics between the treatment groups, a further ANCOVA model was fitted including effects for age, weight and FEV_1_ at baseline. These covariates were not found significant in any model and did not change the interpretation of the results, and so were removed from the final models. A Bayesian approach was taken to use these results to quantify the evidence for a positive effect of the drug for each biomarker. Under the assumption of a noninformative prior for the treatment difference, the posterior probability of a treatment effect greater than zero (percentage of baseline <100%) is equivalent to 1−the one-sided lower p-value (H_1_: µ_Drug_<µ_Placebo_) from this ANCOVA model. An additional analysis including dose as a continuous covariate was also performed. The Pearson correlation coefficient (r) and associated p-value were calculated for percentage of baseline and day 14 values between plasma biomarkers and alveolar macrophage Gal-3 expression. If appropriate, Fisher's exact test was performed for whether a subject had a percentage of baseline <100% for the plasma biomarker and alveolar macrophage Gal-3 expression (yes/no) *versus* treatment (placebo *versus* 10 mg). The analysis is exploratory and there was no pre-planned adjustment for multiple testing; the study was not powered for this. Alveolar macrophage Gal-3 expression was considered the primary exploratory analysis with the pre-selected biomarkers secondary. A Bonferroni correction for multiple testing of the secondary end-points comparing the 10 mg group with placebo gives a critical value of 0.0063. Data are presented as the difference in least square means with 95% confidence interval and p-value unless otherwise stated. All analyses were performed using SAS version 9.4 (SAS Institute, Cary, NC, USA). A concentration–response model was fitted to the alveolar macrophage concentration of TD139 for each subject and the alveolar macrophage surface Gal-3 level (percentage of baseline) using Prism version 8.0 (GraphPad, San Diego, CA, USA) to generate maximum effect (*E*_max_) and median inhibitory concentration (IC_50_) values.

## Results

The enrolment and flow of healthy subjects and IPF patients through part 1 and part 2 of the study is detailed in the CONSORT (Consolidated Standards of Reporting Trials) diagrams (figure 1). Subject disposition and demographics are presented in tables 1 and 2. A small imbalance was observed in age, weight, body mass index and FEV_1_; however, this was found not to impact the analysis results.

Overall, TD139 was considered safe and well tolerated at single doses up to 50 mg and multiple inhaled doses up to 10 mg. In part 1, 28 mild treatment-emergent adverse events (TEAEs) were reported by 15 (41.7%) subjects during the study (table 3). The most commonly occurring TEAE associated with TD139 was mild dysgeusia (distortion of sense of taste) (36.1%), but with no serious tolerability issues. Cough was reported, although the incidence was low (11.1%). There were no other TEAEs of note and in general there were no dose-related trends observed. In addition, there were no clinically significant safety findings or treatment- or dose-related changes observed in biochemistry, haematology, vital signs or 12-lead ECG data. In part 2, there were no treatment- or dose-related trends observed in any TEAE profile, biochemistry, haematology, vital signs or 12-lead ECG data following treatment with TD139. 20 patients had at least one TEAE (table 4); however, there were no withdrawals as a result. Most TEAEs were unrelated to the study drug and only two patients had a TEAE (diarrhoea and dysgeusia/oropharyngeal pain) considered possibly related to the study drug. There was one post-treatment severe TEAE of acute exacerbation of IPF (AE-IPF) triggered by pneumonia reported in the 10 mg TD139 IPF group. This patient received all 14 doses of the study drug with the treatment course recorded as uneventful and pneumonia diagnosed 4 days after the last dose of TD139 following hospital admission. The pneumonia worsened, leading to death 32 days later. Infection was regarded as possibly related to the BAL procedure but unrelated to the study drug by the study investigators.

**TABLE 3:** Summary of treatment-emergent adverse events (TEAEs): part 1 (healthy subjects)

	**TD139**						**Placebo**	**Total**
	**0.15 mg**	**1.5 mg**	**3 mg**	**10 mg**	**20 mg**	**50 mg**		
**TEAEs**	0	0	2	7	7	10	2	28
**Subjects reporting ≥1 TEAE**								
TEAE	0	0	2 (50.0)	3 (75.0)	4 (100.0)	4 (100.0)	2 (16.7)	15 (41.7)
Serious TEAE	0	0	0	0	0	0	0	0
TEAE leading to withdrawal	0	0	0	0	0	0	0	0
**Subjects with TEAE by severity**								
Mild	0	0	2 (50.0)	3 (75.0)	4 (100.0)	4 (100.0)	2 (16.7)	15 (41.7)
**Subjects with TEAE by relationship to study IMP**								
Almost definite	0	0	1 (25.0)	3 (75.0)	4 (100.0)	4 (100.0)	2 (16.7)	14 (38.9)
Probable	0	0	0	0	1 (25.0)	0	0	1 (2.8)
Possible	0	0	0	2 (50.0)	1 (25.0)	3 (75.0)	0	6 (16.7)
Unlikely	0	0	1 (25.0)	0	0	0	0	1 (2.8)

Data are presented as n or n (%). A subject with multiple occurrences of an adverse event is counted only once within each level of severity or relationship. IMP: investigational medicinal product.

**TABLE 4:** Summary of treatment-emergent adverse events (TEAEs): part 2 (idiopathic pulmonary fibrosis patients)

	**TD139**			**Placebo**	**Total**
	**0.3 mg**	**3 mg**	**10 mg**		
**TEAEs**	9	10	13	23	55
**Subjects reporting ≥1 TEAE**					
TEAE	4 (80.0)	5 (100.0)	4 (80.0)	7 (77.8)	20 (83.3)
Serious TEAE	0	0	1 (20.0)	0	1 (4.2)
TEAE leading to withdrawal	0	0	0	0	0
**Subjects with TEAE by severity**					
Mild	2 (40.0)	4 (80.0)	1 (20.0)	4 (44.4)	11 (45.8)
Moderate	2 (40.0)	1 (20.0)	2 (40.0)	3 (33.3)	8 (33.3)
Severe	0	0	1 (20.0)	0	1 (4.2)
**Subjects with TEAE by relationship to study IMP**					
Almost definite	0	0	0	0	0
Probable	0	0	0	0	0
Possible	1 (20.0)	1 (20.0)	0	0	2 (8.3)
Unlikely	1 (20.0)	1 (20.0)	2 (40.0)	2 (22.0)	6 (25.0)
Unrelated	2 (40.0)	3 (60.0)	2 (40.0)	5 (55.6)	12 (50.0)

Data are presented as n or n (%). A subject with multiple occurrences of an adverse event is counted only once within each level of severity or relationship. IMP: investigational medicinal product.

**FIGURE 2:**
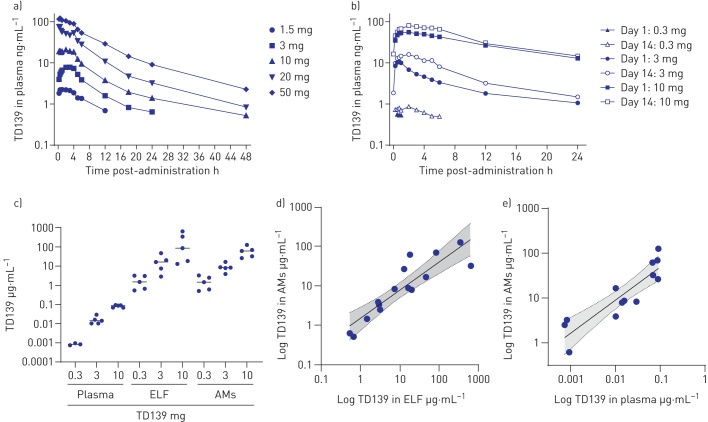
TD139 pharmacokinetics in healthy subjects and idiopathic pulmonary fibrosis (IPF) patients. ELF: epithelial lining fluid; AM: alveolar macrophage. a) Healthy subjects: log-linear mean plasma concentration of TD139 *versus* time over 48 h following a single dose of drug. b) IPF patients: log-linear mean plasma concentration of TD139 *versus* time over 24 h following the first dose of drug on day 1 or the last dose on day 14. c) Log-linear individual measured concentrations (median) in plasma (total), ELF and AMs at 2 h post-administration of 0.3, 3 and 10 mg TD139 on day 14. d) Correlation between concentrations of TD139 in ELF and AMs for all active cohorts on day 14 (r=0.89 (95% CI 0.70–0.96); p<0.0001). e) Correlation between concentrations of TD139 in plasma (total) and AMs for all active cohorts on day 14 (r=0.89 (95% CI 0.65–0.96); p<0.0001). Shading represents 95% CI of the linear fit.

Plasma concentrations of TD139 in healthy subjects and IPF patients are presented in figures 2a and b, respectively, and derived PK parameters are shown in tables 5 and 6. TD139 absorption was nonlinear, increasing with dose and between day 1 and day 14. The PK profile of TD139 in IPF patients in this study was comparable to healthy volunteers; however, the exposure at the highest 10 mg dose was much greater in patients with IPF compared with the same dose in healthy subjects. Thus, a 3.3-fold increase in dose to 10 mg in patients with IPF resulted in a disproportionately high increase in exposure on day 1, with a 5-fold increase in *C*_max_ and a 13-fold increase in AUC_0−inf_. After 14 days of dosing, 10 mg values were 5- and 8-fold greater, respectively, with ∼50% accumulation. With a *T*_1/2_ of ∼8 h and a dosing interval of 24 h, the expected accumulation would be ∼14%. These data suggest that TD139 is retained in the lungs but to a lesser extent in IPF patients compared with healthy subjects and may be saturable, with a slightly greater accumulation in the plasma than expected over time and a much higher systemic exposure with increasing dose. TD139 concentration was assayed in alveolar macrophages derived from patients’ day 14 BAL, in ELF and in plasma (figure 2c). There was a dose-dependent correlation between TD139 concentrations measured between the three compartments (figure 2d and e). The concentration of TD139 in BAL macrophages was between 567- and 1930-fold higher than from systemic exposure at 2 h post-dosing on day 14.

**TABLE 5:** Summary of plasma-derived pharmacokinetics parameters: part 1 (healthy subjects)

	**TD139**					
	**0.15 mg**	**1.5 mg**	**3 mg**	**10 mg**	**20 mg**	**50 mg**
**Subjects**	4	4	4	4	4	4
***C*_max_ ng·mL^−1^**	BLQ	2.37±1.48	8.21±2.67	22.6±8.69	81.1±27.0	125.9±43.3
***T*_1/2_ h**	ND	1.13±0.63	3.00±0.82	1.19±0.94	1.31±1.80	0.81±0.80
**AUC_0−inf_ ng·h·mL^−1^**	ND	17.9±14.0	70.8±22.7	184.4±64.5	543.8±113.6	1161.1±532.0

Data are presented as n or mean±sd. *C*_max_: maximum plasma concentration; *T*_1/2_: plasma half-life; AUC_0–__inf_: area under the curve extrapolated to infinity from dosing time, based on the last observed concentration at 24 h post-dose; BLQ: below limit of quantification (0.5 ng·mL^−1^); ND: not determined due to insufficient data.

**TABLE 6:** Summary of plasma-derived pharmacokinetics parameters: part 2 (idiopathic pulmonary fibrosis patients)

	**TD139**		
	**0.3 mg**	**3 mg**	**10 mg**
**Subjects**	5	5	5
***C*_max_ ng·mL^−1^**			
Day 1	0.55±0.02	10.6±11.1	56.3±19.3
Day 14	0.86±0.25	16.5±8.34	82.8±11.8
***C*_min_ ng·mL^−1^**			
Day 1	BLQ	1.06±0.43	12.9±7.88
Day 14	BLQ	1.48±0.51	14.7±8.64
***T*_1/2_ h**			
Day 1	ND	6.36±2.09	9.69±1.70
Day 14	ND	6.91±1.40	8.20±2.26
**AUC_0−inf_ ng·h·mL^−1^**			
Day 1	ND	69±50	921±479
Day 14	ND	149±47	1150±462

Data are presented as n or mean±sd. *C*_max_: maximum plasma concentration; *C*_min_: plasma concentration at 24 h post-dose; *T*_1/2_: plasma half-life; AUC_0-__inf_: area under the curve extrapolated to infinity from dosing time, based on the last observed concentration at 24 h post-dose; BLQ: below limit of quantification (0.5 ng·mL^−1^); ND: not determined due to insufficient data.

**FIGURE 3:**
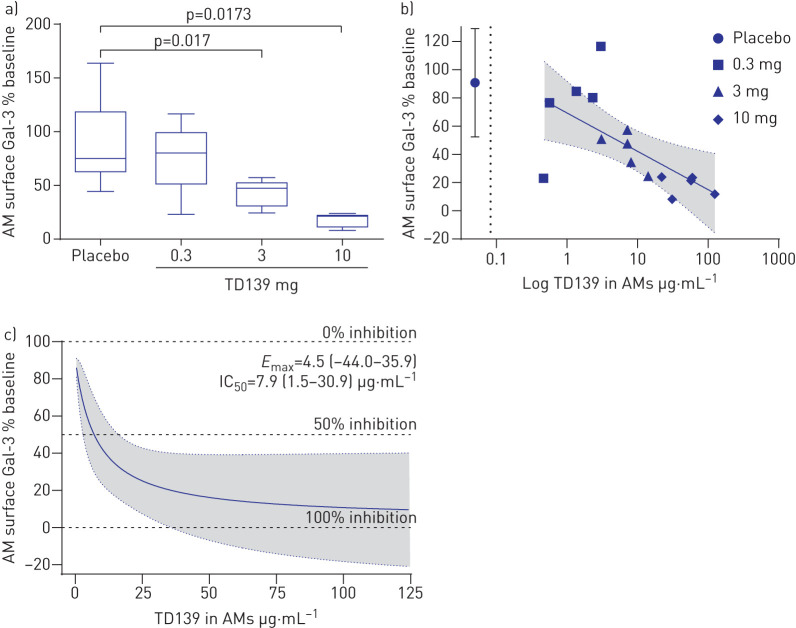
Galectin (Gal)-3 changes in alveolar macrophages (AMs). a) Box plot of TD139 dose-dependent effect on percentage change from baseline in surface macrophage Gal-3 levels. Boxes indicate median and interquartile range; whiskers indicate minimum–maximum range. p-value from ANCOVA model adjusted for baseline. b) Concentration-dependent effect between TD139 in AMs and percentage of baseline in surface macrophage Gal-3, with maximum plasma concentration (*C*_max_) at 10 mg dose (dotted line) shown for comparison (r= −0.63 (95% CI −0.86– −0.18); p=0.011). Shading represents 95% CI of the linear fit. Individual subject data are labelled by dose. c) Fitted maximum effect model of surface macrophage Gal-3 (percentage of baseline) *versus* macrophage TD139 concentration. Maximum effect (*E*_max_, AM surface Gal-3 percentage of baseline) and median inhibitory concentration (IC_50_) values are shown. Shading represents 95% CI of the fitted maximum effect model.

Gal-3 expression on BAL macrophages was reduced by administration of TD139 (figure 3a and supplementary figure S3). The percentage of baseline at day 14 differed between the 3 mg (−38.66% (95% CI −69.59– −7.73%); p=0.017) and 10 mg (−44.63% (95% CI −80.44– −8.81%); p=0.0173) dose groups compared with placebo. A concentration-dependent reduction in cell surface Gal-3 was observed in BAL macrophages, with a near-maximal effect observed in the 10 mg TD139 cohort (figure 3b and c). The concentrations required to induce this effect were several orders of magnitude greater than that present in the systemic circulation (figure 3b).

**FIGURE 4:**
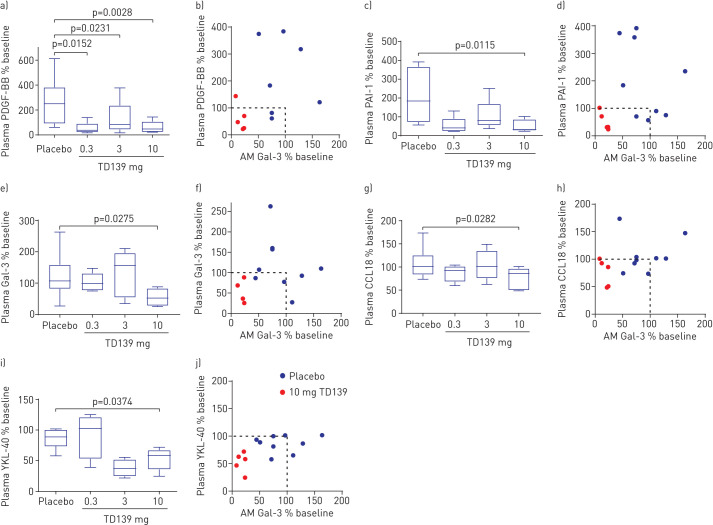
Biomarker changes in plasma. PDGF: platelet-derived growth factor; AM: alveolar macrophage; PAI: plasminogen activator inhibitor; Gal: galectin; CCL: chemokine (C-C motif) ligand. a, c, e, g, i) Percentage of baseline at day 14 for a) PDGF-BB, c) PAI-1, e) Gal-3, g) CCL18 and i) YKL-40 in the 0.3, 3 and 10 mg TD139 dose groups or placebo. Boxes indicate median and interquartile range; whiskers indicate minimum–maximum range. p-value from ANCOVA model adjusted for baseline. b, d, f, h, j) Correlation between percentage of baseline in b) PDGF-BB, d) PAI-1, f) Gal-3, h) CCL18 and j) YKL-40 *versus* the change in lung macrophage Gal-3 expression in the placebo and 10 mg TD139 groups. Dotted line shows no change from baseline. Fisher's exact test for percentage of baseline <100% for plasma Gal-3 and AM Gal-3 expression (yes/no) *versus* treatment group (placebo, 10 mg) p=0.031.

Circulating Gal-3 plasma concentrations (percentage of baseline) were reduced in the highest dose group (10 mg) *versus* placebo (−67.63% (95% CI −126.86– −8.40%); p=0.0275) and correlated with the change in expression of Gal-3 on BAL macrophages at day 14 (figure 4). Fisher's exact test of whether a subject has percentage of baseline <100% for Gal-3 plasma and Gal-3 on BAL macrophages compared for treatment groups found a difference between the 10 mg *versus* placebo groups (p=0.031).

Plasma from patients at day −1 and day 14 was analysed for the expression of a panel of pre-specified inflammatory and fibrosis biomarkers. A disease relevance score was retrospectively assigned to these biomarkers to rank them for importance in IPF progression (table 7 and supplementary table S2). When comparing placebo with the 10 mg TD139 group after adjusting for baseline values, five of the 11 high-relevance plasma markers (PDGF-BB, plasminogen activator inhibitor (PAI)-1, Gal-3, CCL18 and YKL-40) with a link to IPF pathogenesis were reduced (p<0.05). The Bayesian probability that the treatment effect is greater than zero (percentage of baseline <100%) in the 10 mg group is >98% (figure 4, table 7 and supplementary table S2). Another four biomarkers with medium or high relevance ranking (matrix metalloproteinase (MMP)-8, PDGF-AA, hepatocyte growth factor and MMP-1) were impacted by treatment with >90% probability (table 7). Similar results were found when dose was included as a continuous covariate (supplementary table S4). There was also a good correlation between the change in lung Gal-3 concentration and the change in plasma levels of the high-relevance biomarkers in the 10 mg group (figure 4). Overall, the differential impact of TD139 was predominantly on profibrotic *versus* pro-inflammatory markers. In addition, there was no significant change in any biomarker between day −1 and day 14 in the placebo group (table 7).

**TABLE 7:** Summary of statistical analysis of plasma pharmacodynamics biomarkers

**Biomarker**	**Least square means % baseline±sem**				**Difference in least square means** **% baseline (95% CI)^#^**	**Two-sided p-value^#^**	**Bayesian probability of effect^#^**
	**Placebo**	**0.3 mg**	**3 mg**	**10 mg**			
**PDGF-BB**	275.2±42.2	90.5±55.2	103.7±54.1	35.7±54.2	−239.5 (−384.8– −94.2)	0.003	99.86
**PAI-1**	197.6±32.7	84.6±48.8	92.4±44.5	45.8±43.7	−151.8 (−265.3– −38.2)	0.012	99.43
**Gal-3 (BAL)**	81.7±9.0	67.9±12.1	43.0±11.7	37.1±13.5	−44.6 (−80.4– −8.8)	0.017	99.14
**Gal-3**	121.0±15.6	113.9±21.2	119.5±21.3	53.4±23.5	−67.6 (−126.9– −8.4)	0.028	98.63
**CCL18 (PARC)**	109.5±8.8	89.6±11.8	98.9±12.0	74.6±11.8	−35.0 (−65.8– −4.2)	0.028	98.59
**YKL-40 (CHI3L1)**	80.4±7.9	84.1±12.5	50.2±18.6	51.6±10.4	−28.9 (−55.9– −1.9)	0.037	98.13
**MMP-8**	176.9±39.3	138.0±52.8	115.2±52.7	51.5±56.1	−125.4 (−271.3–20.5)	0.088	95.60
**PDGF-AA**	213.3±45.6	61.7±60.6	117.1±62.1	83.6±61.2	−129.7 (−295.3–35.8)	0.117	94.13
**HGF**	110.3±12.6	100.3±17.5	103.2±16.9	73.2±18.8	−37.2 (−85.5–11.2)	0.124	93.79
**MMP-1**	164.2±42.0	67.3±56.3	107.1±59.1	62.1±58.1	−102.1 (−251.4–47.3)	0.169	91.55
**CCL2 (MCP-1)**	161.1±29.6	126.7±39.3	93.7±38.7	89.4±43.5	−71.7 (−187.1–43.7)	0.209	89.55
**CCL5 (RANTES)**	129.9±25.7	62.3±37.1	115.1±35.1	75.8±35.8	−54.1 (−147–38.8)	0.238	88.10
**MIF**	194.1±62.2	123.6±83.6	95.9±83.9	73.9±83.7	−120.2 (−339.2–98.9)	0.265	86.74
**MMP-7**	166.4±44.6	79.3±59.2	82.3±58.4	84.4±58.7	−82.0 (−237.9–73.9)	0.285	85.76
**PTX3**	136.5±41.8	51.8±68.6	74.2±53.1	67.0±56.1	−69.6 (−220.3–81.2)	0.343	82.88
**Osteopontin**	118.1±17.9	106.2±23.9	84±23.7	91.3±23.4	−26.8 (−88.0–34.3)	0.370	81.50
**Periostin**	98.4±3.7	114.0±5.3	99.3±5.2	93.1±5.0	−5.4 (−18.8–8.0)	0.412	79.42
**Gal-1**	106.4±12.4	93.1±16.7	120.2±16.6	89.7±16.6	−16.6 (−60.0–26.7)	0.431	78.45
**IL-13**	105.2±6.6	87.4±8.8	100.4±9.5	97.6±8.8	−7.6 (−30.4–15.2)	0.496	75.22
**SP-D**	114.7±12.0	109.6±16.6	111.7±18.2	118.7±17.3	4.0 (−41.0–49.1)	0.854	42.67
**IL-1ra**	92.0±15.0	93.2±20.3	98.7±20.2	98.0±20.8	6.0 (−47.5–59.4)	0.818	40.89
**TIMP-1**	102.2±4.1	88.6±5.8	107.0±6.0	106.0±5.5	3.8 (−10.6–18.2)	0.583	29.17

PDGF: platelet-derived growth factor; PAI: plasminogen activator inhibitor; Gal: galectin; BAL: bronchoalveolar lavage; CCL: chemokine (C-C motif) ligand; PARC: pulmonary and activation-regulated chemokine; CHI3L1: chitinase-3-like protein 1; MMP: matrix metalloproteinase; HGF: hepatocyte growth factor; MCP: monocyte chemoattractant protein; RANTES: regulated on activation, normal T-cell expressed and secreted; MIF: macrophage migration inhibitory factor; PTX: pentraxin; IL: interleukin; SP-D: surfactant protein D; ra: receptor antagonist; TIMP: tissue inhibitor of metalloproteinases; CXCL: chemokine (C-X-C motif) ligand. For the following biomarkers, the majority of values were below the lower level of quantification and therefore were not analysed: amphiregulin, CCL26 (eotaxin), CXCL1 (GROα), CXCL10 (IP-10), epidermal growth factor, IL-8, IL-10, IL-25, IL-33, interferon-γ and tumour necrosis factor-α. ^#^: results from an ANCOVA model including effects for treatment group and baseline value with the Bayesian probability of effect of the 10 mg TD139 group *versus* placebo shown.

## Discussion

This is the first study to evaluate the pharmacology of a novel therapeutic targeting Gal-3 inhibition in IPF. In addition, it is also the first inhaled therapeutic to be investigated in an IPF clinical study. A large body of evidence points to Gal-3 being an important mediator of fibrosis across multiple organs and pre-clinical work with TD139 has demonstrated the antifibrotic potential of targeting Gal-3 in lung fibrosis [6, 15]. An initial study in healthy subjects (part 1) demonstrated that single doses of TD139 between 0.15 and 50 mg were well tolerated. Reported adverse events were all judged to be mild and inhaled administration of TD139 in this healthy subject study demonstrated a favourable PK profile that supported undertaking a multiple, ascending dose study in individuals with IPF (part 2).

Daily inhaled administration of TD139 for 14 days *via* a dry powder inhaler was safe and well tolerated by patients with IPF, and the adverse events observed were mild in severity. None of the TEAEs resulted in discontinuation of treatment. The SAE in this study was deemed to be a fatal AE-IPF. The subject experienced new respiratory symptoms 2 days after the second BAL and was initially treated for presumed infection but subsequently went on to develop AE-IPF according to the then relevant 2011 criteria [1]. Molyneaux *et al.* [23] have recently reported on the safety of BAL in IPF in a large prospective cohort. However, we are aware that there are published data that indicate that BAL could cause AE-IPF but this is uncommon or rare [24]. Repeat BAL could plausibly increase this risk, *e.g.* if the first BAL induced a “priming” environment that could trigger an AE-IPF with a second BAL. A number of studies, including the current study, have successfully repeated BAL in IPF subjects with a cumulative experience of more than 80 subjects and no reports of procedure-related adverse events [25, 26]. The interval of 14 days between BALs in this study was determined in part to mitigate against this hypothetical situation.

Inhaled administration of TD139 resulted in measurable, dose-dependent levels of the drug in plasma, ELF and alveolar macrophages. The PK profile of TD139 in individuals with IPF was consistent and characterised by low inter- and intra-patient variability. The PK profile in subjects with IPF was comparable to the profile in healthy subjects, although the exposure in IPF was much higher at the 10 mg dose. The AUC_0−inf_ values in IPF patients receiving 10 mg after 14 days were almost equivalent to the 50 mg values in healthy subjects receiving a single dose. Loss of alveolar barrier integrity with increased epithelial permeability is well described in the fibrotic lung [27], which may account for the high systemic levels observed in IPF patients with TD139. The variability of the plasma levels at day 1 was larger than at day 14 for all three dose levels, indicating that patients may achieve a more consistent and reproducible drug exposure as they become accustomed to using the inhaler. Drug levels in the lung determined in ELF and alveolar macrophages showed a strong correlation with plasma levels, allowing a model for achieved lung exposure to be built based on the levels in the systemic circulation. This, combined with the plasma profile observed for TD139, suggested a sustained release of TD139 from the lung into the systemic compartment, demonstrating an extended lung retention. This would be predicted to achieve a prolonged inhibition of Gal-3 in the lung over a 24 h dosing period.

The Gal-3 levels on alveolar macrophages were inhibited by TD139 in a concentration-dependent manner, with the PK/PD relationship defined in the target compartment and the 10 mg dose causing almost maximal inhibition. In addition, the highest dose of TD139 also decreased circulating Gal-3, which correlated with a reduction in several fibrosis-related biomarkers. Four highly relevant disease biomarkers, *i.e.* PDGF-BB [28–31], CCL18 [32, 33], PAI-1 [34–38] and YKL-40 [39–41], have been shown to have prognostic significance in IPF and have well-described effects on myofibroblast activity *in vitro*. These biomarkers were reduced from baseline for the 10 mg dose group compared with placebo and offer a less invasive measure of PD effects going forward into the next phase of clinical development for TD139. The partial reduction in BAL macrophage Gal-3 in the 0.3 and 3 mg groups was associated with a reduction in some biomarkers, *e.g.* PDGF-BB in plasma. Due to the pleiotropic nature of Gal-3 in fibrotic disease and the many signalling pathways it influences, it could be hypothesised that some could be more sensitive to Gal-3 modulation than others. This could result in maximal downstream inhibition of biomarkers such as PDGF-BB with only a partial inhibition of Gal-3.

In a *post hoc* analysis of the ASCEND and CAPACITY trials, both YKL-40 and CCL18 were prognostic for progression in the test cohort but only CCL18 was consistently prognostic for a change in FVC in both the test and replication cohorts [42]. However, of note there was no association between pirfenidone treatment and the longitudinal concentration of any biomarker [42]. In another study comparing treatment-naive IPF patients with those on antifibrotic treatment (pirfenidone or nintedanib), CA-125, CXCL13, MMP-7, YKL-40 and osteopontin predicted differential transplant-free survival in treated patients but at higher thresholds than treatment-naive individuals [43]. There is therefore substantial evidence that several biomarkers are related to disease severity and prognosis, particularly YKL-40 and CCL18, but no conclusive study that relates biomarker changes in currently approved antifibrotic therapy to survival.

The limitations of this study are the small sample size, short duration of treatment and mild severity of disease. It is therefore encouraging that TD139 has demonstrated evidence of shifts in these biomarkers despite these limitations.

In conclusion, inhaled dosing of TD139 for 14 days to individuals with IPF has been shown to be safe and well tolerated. TD139 effectively engages with Gal-3 in the alveolar space and is associated with favourable shifts in mediators that plausibly drive lung fibrosis. These data provide the information necessary for selecting the optimal dosing strategy for TD139, and strongly support performing longer and larger trials in individuals with IPF to assess antifibrotic efficacy and the effect on lung function. A phase 2b study with TD139 in individuals with IPF is now underway (GALACTIC-1; ClinicalTrials.gov: NCT03832946).

## Supplementary material

10.1183/13993003.02559-2020.Supp1 **Please note:** supplementary material is not edited by the Editorial Office, and is uploaded as it has been supplied by the author.
Supplementary methods and tables ERJ-02559-2020.SUPPLEMENT
Supplementary figures ERJ-02559-2020.FIGURES

## Shareable PDF

10.1183/13993003.02559-2020.Shareable1 This one-page PDF can be shared freely online.
Shareable PDF ERJ-02559-2020.Shareable
