# Supplementary material for: Target inhibition of galectin-3 by inhaled TD139 in patients with idiopathic pulmonary fibrosis
Source: Eur Respir J. 2021 May 27;57(5):2002559. doi: 10.1183/13993003.02559-2020 (PMC8156151; doi:10.1183/13993003.02559-2020)
Supplement: Supplementary file 1 [file ERJ-02559-2020.SUPPLEMENT.pdf]

SUPPLEMENTAL

METHODS

Flow cytometry

BAL cells were separated from fluid by centrifugation and BAL cells were collected, stained with antibodies then fixed before analysis using a LSRFortessa™ flow cytometer (Becton Dickinson, NJ, USA). Data were analysed using FlowJo software (Becton Dickinson, NJ, USA). After exclusion of doublets and debris a sequential gating strategy was employed to identify cell populations based on surface marker expression. The antibodies used were Gal-3-FITC, CD19-PE, HLA-DR PEcy7, CD14-APC, CD16-pacific-blue, CD11b perCPcy5.5, CD62L-PE-cy7, CD4-AF488, CD56-PE, CD147-perCPcy5.5, CD25-APC, CD8-AF700, CD44-PAC blue, CD3-PE-cy7, CD163-APC, CD206-PE, CD209-perCP5.5 all from Biolegend except Gal-3-FITC which was from Cedarlane. Macrophages were identified as having high side scatter properties and HLA-DR positivity. Gal-3 expression (mean fluorescence intensity, MFI) was determined from the macrophage gate. Macrophage subsets were classed as M_1, M_{2a} and M_{2c} based on the relative expression of CD206 and CD163 as follows; (M_1 $CD206^-/CD163^-$, M_{2a} $CD206^+/CD163^-$, M_{2c} $CD206^+/CD163^+$). In the HLA-DR-ve gate the relative % of neutrophils ($CD19^-/CD16^+$), B cells ($CD19^+$), natural killer (NK) cells ($CD56^+$), NK T-cells (NKT) cells ($CD56^+/CD3^+$), CD4 T cells ($CD3^+/CD4^+$) and CD8 T cells ($CD3^+/CD8^+$) and regulatory T cells (Tregs) ($CD3^+/CD4^+/CD25^+/CD147^-$) were recorded.

Plasma pharmacodynamic biomarkers

The following pre-specified biomarkers were evaluated in samples of plasma based on the basis that these proteins are known to be involved in Gal-3 pathways and/or suggested to be putative biomarkers in IPF: amphiregulin, chemokine (C-C motif) ligand 2 (CCL2) [monocyte chemoattractant protein 1], CCL5 [Regulated on Activation, Normal T Expressed and Secreted], CCL18 [pulmonary and activation-regulated chemokine], CCL26 [eotaxin], chemokine (C-X-C motif) ligand 1 (CXCL1) [growth-regulated oncogene-α], CXCL8 [interleukin-8 (IL-8)], CXCL10 [IP-10], epidermal growth factor (EGF), Galectin-1 (Gal-1), Gal-3, hepatocyte growth factor (HGF), interferon-γ (IFNγ), IL-1 receptor antagonists (IL-1ra), IL-10, IL-12, IL-13, IL-25, IL-33, macrophage migration inhibitory factor (MIF), matrix metalloproteinase-1 (MMP-1), MMP-7, MMP-8, osteopontin, periostin, plasminogen activator inhinitor-1 (PAI-1), pentraxin-3 (PTX3), surfactant protein D (SP-D), tissue inhibitor of metalloproteinases 1 (TIMP1), tumor necrosis factor-α (TNFα), vascular endothelial growth factor (VEGF) and YKL-40 [chitinase-3-like protein 1]. A disease relevance score was retrospectively assigned to these biomarkers to rank them for importance in IPF progression (supplementary table S2).

RESULTS

BAL cell classification and quantification

HLA-DR positive alveolar macrophages were subclassified into M_1, M_{2a} and M_{2c} based on CD163 and CD206 expression (supplemental figure S2). There was a trend for an increase in the relative percentage of M_{2c} cells and corresponding decrease in the percentage of M_{2a} cells following treatment with TD139 in the high dose group. Although baseline frequencies showed some variation between groups there was no change in the abundance or frequencies of CD4+ or $CD8^+$ T cells, Tregs, NK, NKT, B cells, neutrophils or monocytes or the percentage of inducible or resident monocyte populations with TD139 in any dose groups (supplemental figure S4).

SUPPLEMENTAL TABLE S1 Inclusion and exclusion criteria

Part 1 – Healthy subjects

Inclusion criteria

- Healthy male subjects aged between 18 and 55 years of age.
- Male subject willing to use a condom, if applicable (unless anatomically sterile or where abstaining from sexual intercourse is in line with the preferred and usual lifestyle of the subject) from the Day 1 dose of study medication until 3 months afterwards.
- Subject with a body weight of at least 50 kg and a body mass index (BMI) within the range of 18-35 kg/m^2. BMI = Body weight (kg) / [Height (m)]^2.
- Subject with no clinically significant abnormal serum biochemistry, haematology and urine examination values within 28 days of the Day 1 dose of study medication.
- Subject with a negative urinary drugs of abuse screen, determined within 28 days of the Day 1 dose of study medication, (N.B. a positive alcohol result may be repeated at the discretion of the Investigator).
- Subject with negative human immunodeficiency virus (HIV) and hepatitis B surface antigen (Hep B) and hepatitis C virus antibody (Hep C) results.
- Subject with no clinically significant abnormalities in 12-lead ECG determined within 28 days of the Day 1 dose of study medication.
- Subjects were non-smokers or former smokers (having ceased smoking for at least 6 months).
- Subjects with no clinically significant impairment in oxygen saturation.
- Subject satisfied a medical examiner about their fitness to participate in the study.
- Subject provided written informed consent to participate in the study.
- Subject was available to complete the study (including all follow up visits).
- Healthy male non-smoker or a former smoker (having ceased smoking for at least 6 months) aged between 18 and 55 years, weighing ≥ 50 kg with a body mass index (BMI) of 18-35 kg/m2.
- No history of hypersensitivity (anaphylaxis, angioedema) to any drug, or allergy, significant adverse reaction to nicotine, cholinergic drugs, drugs with a similar chemical structure, or drugs similar to the investigational drug.

Exclusion criteria

- A clinically significant illness or surgery within 8 weeks prior to the Day 1 dose of study medication.
- Significant medical history that, in the Investigator's opinion, may have adversely affected participation.
- History of allergy or significant adverse reaction to drugs similar to the investigational drug, to nicotine, or to cholinergic drugs or to any drugs with a similar chemical structure.
- History of hypersensitivity (anaphylaxis, angioedema) to any drug.
- Use of any drug known to induce or inhibit hepatic drug metabolism, within 30 days prior to the Day 1 dose of study medication.
- Use of medications known to prolong QT/QTc interval within 14 days prior to the Day 1 dose of study medication.

- Any clinically significant findings of physical examination or laboratory findings at screening.
- A clinically significant history of drug or alcohol abuse.
- Receipt of regular/over-the-counter medication within 14 days of the Day 1 dose of study medication that may have had an impact on the safety and objectives of the study (at the Investigator's discretion).
- Evidence of renal, hepatic, central nervous system, respiratory, cardiovascular or metabolic dysfunction.
- Inability to communicate well with the Investigator (i.e., language problem, poor mental development or impaired cerebral function).
- Participation in a New Chemical Entity clinical study within the previous 4 months or a marketed drug clinical study within the previous 3 months. (N.B. washout period between studies is defined as the period of time elapsed between the last dose of the previous study and the first dose of the next study).
- Donation of 450 mL or more blood within the previous 3 months.

Part 2 – IPF patients

Inclusion criteria

- males or females of non-childbearing potential with IPF.
- age between 45 and 85 years of age.
- FVC ≥ 45% predicted and an FEV1/FVC ratio ≥ 0.7.
- Oxygen saturation >90% by pulse oximetry while breathing ambient air at rest.
- diffusing capacity (DLCO) >25%.
- a clinical diagnosis consistent with IPF prior to screening (based on ATS/ERS/JRS/ALAT consensus criteria confirmed at a multidisciplinary team meeting where the HRCT findings will have been discussed with a radiologist).
- able to undergo bronchoalveolar lavage (BAL).
- able to provide written informed consent to participate in the study.
- human immunodeficiency virus (HIV) and hepatitis B surface antigen (Hep B) and hepatitis C virus antibody (Hep C) negative confirmed at screening.
- no clinically significant abnormalities in 12-lead electrocardiogram (ECG) determined within 28 days of the first dose.
- negative urinary drugs of abuse screen, determined within 28 days of the first dose.

Exclusion criteria

- Any condition that makes the patient at unacceptable risk for bronchoscopy.
- Active cigarette smoking (defined as smoking more than 3 cigarettes daily within the last 6 months).
- Presence of a significant co-morbidity felt to limit life expectancy to less than 12 months.
- HRCT pattern showing emphysema more than the extent of fibrosis of the lung area conducted within 12 months of Day 1.
- Evidence of renal, hepatic, central nervous system, or metabolic dysfunction.
- Evidence of poorly controlled diabetes mellitus (defined as a HbA1c of > 59 mmol/mol [7.5%]).
- Use of systemic immunosuppressants within 30 days of dosing.

- Subjects currently receiving oral corticosteroids, cytotoxic drugs (e.g. chlorambucil, azathioprine, cyclophosphamide, methotrexate), antifibrotic drugs (e.g. pirfenidone), vasodilator therapies for pulmonary hypertension (e.g. bosentan), unapproved (e.g. INF-γ, penicillamine, cyclosporine, mycophenolate) and/or investigational therapies for IPF or administration of such therapies within 4 weeks of initial screening. A current inhaled steroid dose of ≤1000 micrograms beclomethasone dipropionate (BDP) equivalent per day is acceptable if the dose is anticipated to remain stable during the study.
- History of malignancy, including carcinoma during the preceding five years.
- History of, or current asthma.

Participation in a clinical study of an unlicensed drug in the previous 4 months, or a marketed drug study within the previous 3 months.

N.B. washout period between trials defined as the period of time elapsed between the last dose of the previous study and the first dose of the next study.

SUPPLEMENTAL TABLE S2 – Plasma pharmacodynamic biomarker relevance criteria

**Biomarker**	***Bayesian probability of effect (10 mg)**	**Disease Relevance Criteria**				
		**1**	**2**	**3**	**4**	**5**
**PDGF-BB**	99.86	(1)	(2,3)	(2,4)	(2,3)	(5)
**PAI-1**	99.43	(6,7)	(8-10)	(7,8,11)	(8,9)	(12)
**Galectin-3**	98.63	(13)	(13,14)	(13,14)	(13,14)	
**CCL18 (PARC)**	98.59	(15)	(15,16)	(17)	(15)	(18)
**YKL-40 (CHI3L1)**	98.13	(19)	(19-21)	(19)	(19-21)	
**MMP-8**	95.60		(22)	(22)	(22)	
**PDGF-AA**	94.13	(1)	(23)	(4)	(23)	(5)
**HGF**	93.79			(24)		
**MMP-1**	91.55		(25,26)	(25)	(25,26)	
**MIF**	<90.0		(27)	(27,28)	(27)	
**CCL2 (MCP-1)**	<90.0		(29)	(30)	(29)	(18)
**TIMP1**	<90.0		(26,31)	(26)	(26,31)	(5)
**MMP-7**	<90.0	(25)	(25,26)	(25)	(25,26)	
**IL-13**	<90.0		(32)	(33)	(32)	(18)
**SP-D**	<90.0	(34)	(34,35)	(35)	(34,35)	
**CCL5 (RANTES)**	<90.0		(36)	(36)	(36)	
**Osteopontin**	<90.0		(37)	(37)	(37)	
**Galectin-1**	<90.0		(38,39)	(39)	(38,39)	
**Periostin**	<90.0		(40,41)	(40,42)	(40,41)	
**Pentraxin-3 (PTX-3)**	<90.0		(43)		(44)	
**IL-1ra**	<90.0			(45)		

The plasma biomarkers measured were given a disease relevance score by applying 5 criteria: (1) correlation with disease outcome, (2) validated fibrosis effector mechanism *in vitro* and *in vivo*, (3) elevated systemically or in the lung of IPF patients, (4) expressed in key disease cells (fibroblasts/macrophages) and (5) link to the mechanism of action of Nintedanib (approved IPF treatment). A biomarker was considered high

relevance if 4-5 criteria were met; medium relevance if 2-3 criteria met, and low if 0-1 criteria met (see supplemental references for supporting evidence). Results from an analysis of covariance model including effects for treatment group and baseline value with the Bayesian probability of effect of 10 mg TD139 group *vs.* placebo are shown*.

SUPPLEMENTAL TABLE S3 Plasma pharmacodynamic biomarker raw data

Biomarker	Day	Mean (SD) pg/ml			
		**Placebo**	**0.3 mg**	**3 mg**	**10 mg**
**CCL2 (MCP-1)**	1	111.3 (79.75)	112.2 (37.78)	164.5 (67.02)	357.1 (375.36)
	14	148.7 (85.53)	133.8 (15.06)	137.6 (63.21)	270.3 (348.45)
**CCL5 (RANTES)**	1	81617.7 (82526.36)	125883.5 (63253.82)	53815.5 (21806.74)	41872.9 (21417.78)
	14	83297.5 (87964.32)	47658.1 (27017.8)	75675.6 (92820.57)	33994.2 (29435.9)
**CCL18 (PARC)**	1	88328.6 (39842.99)	91072.2 (15471.37)	68752.8 (17747.82)	80185.3 (38709.52)
	14	88335.6 (35582.91)	79733.8 (25166.88)	73022.7 (33487.12)	53456.8 (11339.99)
**Galectin-1**	1	56708.6 (28698.58)	48231.4 (20322.42)	53988.6 (11006.04)	55630.8 (24962.77)
	14	52118.8 (16994.43)	41996.4 (7357.87)	60416.7 (17348.65)	48409.7 (21339.06)
**Galectin-3**	1	10900.4 (10123.84)	12934.3 (3948.52)	8269.5 (2684.8)	10447.8 (6157.76)
	14	8938.9 (3208.78)	13290.4 (4951.24)	9298.5 (3499.54)	4486.3 (697.25)
**Galectin-3 (BAL)**	1	16475.9 (5917.25)	15310.4 (15593.37)	19995.8 (8088)	30449.8 (6574.47)
	14	13590.2 (5754.29)	7794.8 (2990.48)	8058.6 (3001.05)	5237 (2033.86)
**HGF**	1	143.1 (66.58)	105.7 (15.28)	140.3 (29.60)	244.7 (172.4)
	14	144.2 (49.74)	109.8 (23.24)	159.1 (110.2)	131.2 (44.47)
**IL-1ra**	1	1335.8 (2143.31)	580.8 (340.16)	764.4 (216.85)	2401.1 (3495.04)
	14	559.4 (221.94)	548.3 (279.69)	709.8 (338.05)	1992.9 (2627.15)
**IL-13**	1	1350.5 (235.89)	1385.7 (253.53)	1784.3 (751.45)	1368.5 (178.84)
	14	1458.1 (493.82)	1219.4 (155.7)	1476.6 (140.99)	1358.8 (149.34)
**MIF**	1	54503.1 (54799.79)	69021.4 (27636.58)	47342.3 (30164.83)	70206.1 (64466.35)
	14	53472.9 (44175.64)	65871.3 (42284.53)	38884 (35236.82)	39660.6 (57202.1)
**MMP-1**	1	1189.2 (1137.8)	1187.8 (1196.68)	1858.7 (798.94)	741.6 (237.64)
	14	1842.5 (2072.58)	535.1 (449.22)	1494.4 (647.67)	537.5 (257.55)
**MMP-7**	1	3951.3 (8150.31)	560.7 (259.3)	1991.7 (2160.04)	1171.7 (887.93)
	14	6056.7 (14116.38)	447.6 (205.21)	1849.2 (2244.12)	1130.6 (939.62)
**MMP-8**	1	10716.6 (28261)	1412.8 (422.24)	1969.4 (844.47)	139102.5 (306988.65)
	14	2222.3 (1575.24)	1911.5 (1577.82)	2263.3 (2428.78)	44436.7 (97465.67)

**Osteopontin**	1	68739.2 (57730.49)	34241.8 (13563.92)	38808.4 (22367.52)	60286 (48443.67)
	14	68125.8 (74480.11)	37756 (20451.51)	31032.4 (13230.48)	48630.5 (37737.51)
**PAI-1**	1	50298.9 (43195.44)	97468.4 (52279.19)	41170 (9420.67)	50442.3 (30219.02)
	14	80727.1 (55809.77)	45736.7 (29863.26)	45744.4 (44222.1)	23122.2 (12659.84)
**PDGF-AA**	1	2140.6 (1391.8)	1996.4 (1004.22)	4207 (3264.35)	4019.9 (1276.63)
	14	4104 (3200.22)	1299.7 (754.57)	4075.9 (5511.46)	2556.7 (987.79)
**PDGF-BB**	1	2760.4 (2897.46)	3624 (2279.74)	1791.6 (912.36)	1749.9 (659.84)
	14	3667.3 (2678.96)	1519 (1034.92)	2201.9 (2935.52)	1131.4 (1190.78)
**Periostin**	1	134749.5 (23951.64)	109819 (15283.82)	143649.3 (21734.75)	117090.8 (31288.75)
	14	131484 (24870.01)	125952.3 (17487.63)	142019.1 (30390.08)	108663.7 (24686.82)
**Pentraxin-3 (PTX3)**	1	1379 (2796.23)	170.3 (335.06)	938.4 (738.13)	7055.8 (13193.33)
	14	480.4 (402.83)	116.3 (214.7)	629.9 (581.55)	2534.7 (4451.2)
**SP-D**	1	58685.4 (33447.61)	35595.9 (11189.97)	84308.4 (38808.22)	28901.4 (12165.25)
	14	68522 (43801.19)	38987.4 (10734.36)	91473.6 (43437.58)	30347 (3552.88)
**TIMP1**	1	44543.7 (8450.21)	38898.1 (3364.27)	52018.3 (1712.3)	43880.4 (14514.68)
	14	44090.5 (6162.09)	38084.3 (4735.81)	49880.6 (1032.27)	45458.7 (11908.04)
**YKL-40 (CHI3L1)**	1	141225.5 (100517.87)	50518.5 (26360.63)	417206.4 (144925.81)	146407.5 (30068.05)
	14	110951.3 (78338.33)	45140.2 (34041.85)	146819.5 (40114.19)	79975.5 (38590.77)

For the following biomarkers, the majority of values were below the lower level of quantification and therefore were not analysed: amphiregulin, CCL26 (eotaxin), CXCL1 (GROα), CXCL10 (IP-10), EGF, IL-8, IL-10, IL-25, IL-33, IFNγ and TNFα. BAL, bronchoalveolar lavage.

SUPPLEMENTAL TABLE S4 Plasma pharmacodynamic biomarker analysis including dose as a continuous covariate

Dependent	Estimate	SE	t-statistic	p-value
PDGF-BB	-18.06	7.43	-2.43	0.024
PAI-1	-12.25	5.42	-2.26	0.035
Galectin-3 (BAL)	-4.19	1.72	-2.44	0.024
Galectin-3	-6.45	2.60	-2.48	0.022
CCL18 (PARC)	-2.88	1.39	-2.08	0.050
YKL-40 (CHI3L1)	-2.88	1.20	-2.41	0.026
MMP-8	-11.55	6.37	-1.81	0.084
PDGF-AA	-8.04	7.91	-1.02	0.321
HGF	-3.44	2.15	-1.6	0.125
MMP-1	-7.11	6.69	-1.06	0.301
CCL2 (MCP-1)	-6.35	5.18	-1.23	0.234
CCL5 (RANTES)	-3.93	4.35	-0.9	0.376
MIF	-9.92	9.51	-1.04	0.309
MMP-7	-5.22	6.87	-0.76	0.456
PTX-3	-5.14	6.72	-0.76	0.455
Osteopontin	-2.40	2.67	-0.9	0.379
Periostin	-1.15	0.63	-1.81	0.084
Galectin-1	-1.17	1.94	-0.6	0.552
IL-13	-0.17	1.04	-0.16	0.876
SP-D	0.56	1.84	0.31	0.763
IL-1ra	0.60	2.32	0.26	0.799
TIMP-1	0.82	0.69	1.2	0.244

SUPPLEMENTAL REFERENCES

1. Zhu, X., Fang, X., Chen, W., Han, F., Huang, Z., Luo, B., Gu, P., Zhang, L., Qiu, W., Zeng, Y., Rui, W., and Yi, X. (2017) Lower expression of platelet derived growth factor is associated with better overall survival rate of patients with idiopathic nonspecific interstitial pneumonia. *Journal of thoracic disease* **9**, 519-528
2. Hetzel, M., Bachem, M., Anders, D., Trischler, G., and Faehling, M. (2005) Different effects of growth factors on proliferation and matrix production of normal and fibrotic human lung fibroblasts. *Lung* **183**, 225-237
3. Kishi, M., Aono, Y., Sato, S., Koyama, K., Azuma, M., Abe, S., Kawano, H., Kishi, J., Toyoda, Y., Okazaki, H., Ogawa, H., Uehara, H., and Nishioka, Y. (2018) Blockade of platelet-derived growth factor receptor-beta, not receptor-alpha ameliorates bleomycin-induced pulmonary fibrosis in mice. *PloS one* **13**, e0209786
4. Allen, J. T., and Spiteri, M. A. (2002) Growth factors in idiopathic pulmonary fibrosis: relative roles. *Respir Res* **3**, 13
5. Wollin, L., Wex, E., Pautsch, A., Schnapp, G., Hostettler, K. E., Stowasser, S., and Kolb, M. (2015) Mode of action of nintedanib in the treatment of idiopathic pulmonary fibrosis. *Eur Respir J* **45**, 1434-1445
6. Ban, C., Wang, T., Zhang, S., Xin, P., Liang, L., Wang, C., and Dai, H. (2017) Fibrinolytic system related to pulmonary arterial pressure and lung function of patients with idiopathic pulmonary fibrosis. *Clin Respir J* **11**, 640-647
7. Kotani, I., Sato, A., Hayakawa, H., Urano, T., Takada, Y., and Takada, A. (1995) Increased procoagulant and antifibrinolytic activities in the lungs with idiopathic pulmonary fibrosis. *Thromb Res* **77**, 493-504
8. Liu, R. M. (2008) Oxidative stress, plasminogen activator inhibitor 1, and lung fibrosis. *Antioxid Redox Signal* **10**, 303-319
9. Ghosh, A. K., and Vaughan, D. E. (2012) PAI-1 in tissue fibrosis. *Journal of cellular physiology* **227**, 493-507
10. Flevaris, P., and Vaughan, D. (2017) The Role of Plasminogen Activator Inhibitor Type-1 in Fibrosis. *Seminars in thrombosis and hemostasis* **43**, 169-177
11. Menou, A., Duitman, J., and Crestani, B. (2018) The impaired proteases and anti-proteases balance in Idiopathic Pulmonary Fibrosis. *Matrix Biol* **68-69**, 382-403
12. Knuppel, L., Ishikawa, Y., Aichler, M., Heinzelmann, K., Hatz, R., Behr, J., Walch, A., Bachinger, H. P., Eickelberg, O., and Staab-Weijnitz, C. A. (2017) A Novel Antifibrotic Mechanism of Nintedanib and Pirfenidone. Inhibition of Collagen Fibril Assembly. *Am J Respir Cell Mol Biol* **57**, 77-90
13. Mackinnon, A. C., Gibbons, M. A., Farnworth, S. L., Leffler, H., Nilsson, U. J., Delaine, T., Simpson, A. J., Forbes, S. J., Hirani, N., Gauldie, J., and Sethi, T. (2012) Regulation of transforming growth factor-beta1-driven lung fibrosis by galectin-3. *Am J Respir Crit Care Med* **185**, 537-546
14. Nishi, Y., Sano, H., Kawashima, T., Okada, T., Kuroda, T., Kikkawa, K., Kawashima, S., Tanabe, M., Goto, T., Matsuzawa, Y., Matsumura, R., Tomioka, H., Liu, F. T., and Shirai, K. (2007) Role of galectin-3 in human pulmonary fibrosis. *Allergology international : official journal of the Japanese Society of Allergology* **56**, 57-65
15. Prasse, A., Probst, C., Bargagli, E., Zissel, G., Toews, G. B., Flaherty, K. R., Olschewski, M., Rottoli, P., and Muller-Quernheim, J. (2009) Serum CC-chemokine ligand 18

concentration predicts outcome in idiopathic pulmonary fibrosis. *Am J Respir Crit Care Med* **179**, 717-723
16. Neighbors, M., Cabanski, C. R., Ramalingam, T. R., Sheng, X. R., Tew, G. W., Gu, C., Jia, G., Peng, K., Ray, J. M., Ley, B., Wolters, P. J., Collard, H. R., and Arron, J. R. (2018) Prognostic and predictive biomarkers for patients with idiopathic pulmonary fibrosis treated with pirfenidone: post-hoc assessment of the CAPACITY and ASCEND trials. *Lancet Respir Med* **6**, 615-626
17. Hamai, K., Iwamoto, H., Ishikawa, N., Horimasu, Y., Masuda, T., Miyamoto, S., Nakashima, T., Ohshimo, S., Fujitaka, K., Hamada, H., Hattori, N., and Kohno, N. (2016) Comparative Study of Circulating MMP-7, CCL18, KL-6, SP-A, and SP-D as Disease Markers of Idiopathic Pulmonary Fibrosis. *Dis Markers* **2016**, 4759040
18. Wollin, L., Distler, J. H. W., Redente, E. F., Riches, D. W. H., Stowasser, S., Schlenker-Herceg, R., Maher, T. M., and Kolb, M. (2019) Potential of nintedanib in treatment of progressive fibrosing interstitial lung diseases. *Eur Respir J* **54**
19. Korthagen, N. M., van Moorsel, C. H. M., Barlo, N. P., Ruven, H. J. T., Kruit, A., Heron, M., van den Bosch, J. M. M., and Grutters, J. C. (2011) Serum and BALF YKL-40 levels are predictors of survival in idiopathic pulmonary fibrosis. *Respiratory medicine* **105**, 106-113
20. Letuve, S., Kozhich, A., Arouche, N., Grandsaigne, M., Reed, J., Dombret, M. C., Kiener, P. A., Aubier, M., Coyle, A. J., and Pretolani, M. (2008) YKL-40 is elevated in patients with chronic obstructive pulmonary disease and activates alveolar macrophages. *J Immunol* **181**, 5167-5173
21. Furuhashi, K., Suda, T., Nakamura, Y., Inui, N., Hashimoto, D., Miwa, S., Hayakawa, H., Kusagaya, H., Nakano, Y., Nakamura, H., and Chida, K. (2010) Increased expression of YKL-40, a chitinase-like protein, in serum and lung of patients with idiopathic pulmonary fibrosis. *Respiratory medicine* **104**, 1204-1210
22. Craig, V. J., Zhang, L., Hagood, J. S., and Owen, C. A. (2015) Matrix metalloproteinases as therapeutic targets for idiopathic pulmonary fibrosis. *Am J Respir Cell Mol Biol* **53**, 585-600
23. McGowan, S. E., and McCoy, D. M. (2013) Platelet-derived growth factor-A regulates lung fibroblast S-phase entry through p27(kip1) and FoxO3a. *Respir Res* **14**, 68
24. Ziora, D., Adamek, M., Czuba, Z., Jastrzębski, D., Zeleznik, K., Kasperczyk, S., Kozielski, J., and Krol, W. Increased Serum Hepatocyte Growth Factor (HGF) Levels in Patients with Idiopathic Pulmonary Fibrosis (IPF) or Progressive Sarcoidosis
J Mol Biomark Diagn **5**, 1000167
25. Rosas, I. O., Richards, T. J., Konishi, K., Zhang, Y., Gibson, K., Lokshin, A. E., Lindell, K. O., Cisneros, J., Macdonald, S. D., Pardo, A., Sciurba, F., Dauber, J., Selman, M., Gochuico, B. R., and Kaminski, N. (2008) MMP1 and MMP7 as potential peripheral blood biomarkers in idiopathic pulmonary fibrosis. *PLoS Med* **5**, e93
26. Todd, J. L., Vinisko, R., Liu, Y., Neely, M. L., Overton, R., Flaherty, K. R., Noth, I., Newby, L. K., Lasky, J. A., Olman, M. A., Hesslinger, C., Leonard, T. B., Palmer, S. M., Belperio, J. A., and investigators, I.-P. R. (2020) Circulating matrix metalloproteinases and tissue metalloproteinase inhibitors in patients with idiopathic pulmonary fibrosis in the multicenter IPF-PRO Registry cohort. *BMC Pulm Med* **20**, 64
27. Olivieri, C., Bargagli, E., Inghilleri, S., Campo, I., Cintorino, M., and Rottoli, P. (2016) Macrophage migration inhibitory factor in lung tissue of idiopathic pulmonary fibrosis patients. *Exp Lung Res* **42**, 263-266

28. Bargagli, E., Olivieri, C., Nikiforakis, N., Cintorino, M., Magi, B., Perari, M. G., Vagaggini, C., Spina, D., Prasse, A., and Rottoli, P. (2009) Analysis of macrophage migration inhibitory factor (MIF) in patients with idiopathic pulmonary fibrosis. *Respir Physiol Neurobiol* **167**, 261-267
29. Yang, J., Agarwal, M., Ling, S., Teitz-Tennenbaum, S., Zemans, R. L., Osterholzer, J. J., Sisson, T. H., and Kim, K. K. (2020) Diverse Injury Pathways Induce Alveolar Epithelial Cell CCL2/12 Which Promotes Lung Fibrosis. *Am J Respir Cell Mol Biol*
30. Shinoda, H., Tasaka, S., Fujishima, S., Yamasawa, W., Miyamoto, K., Nakano, Y., Kamata, H., Hasegawa, N., and Ishizaka, A. (2009) Elevated CC chemokine level in bronchoalveolar lavage fluid is predictive of a poor outcome of idiopathic pulmonary fibrosis. *Respiration* **78**, 285-292
31. Dong, J., and Ma, Q. (2019) In Vivo Activation and Pro-Fibrotic Function of NF-kappaB in Fibroblastic Cells During Pulmonary Inflammation and Fibrosis Induced by Carbon Nanotubes. *Frontiers in pharmacology* **10**, 1140
32. Hashimoto, S., Gon, Y., Takeshita, I., Matsumoto, K., Maruoka, S., and Horie, T. (2001) Transforming growth Factor-beta1 induces phenotypic modulation of human lung fibroblasts to myofibroblast through a c-Jun-NH2-terminal kinase-dependent pathway. *Am J Respir Crit Care Med* **163**, 152-157
33. Park, S. W., Ahn, M. H., Jang, H. K., Jang, A. S., Kim, D. J., Koh, E. S., Park, J. S., Uh, S. T., Kim, Y. H., Park, J. S., Paik, S. H., Shin, H. K., Youm, W., and Park, C. S. (2009) Interleukin-13 and its receptors in idiopathic interstitial pneumonia: clinical implications for lung function. *J Korean Med Sci* **24**, 614-620
34. Ikeda, K., Shiratori, M., Chiba, H., Nishikiori, H., Yokoo, K., Saito, A., Hasegawa, Y., Kuronuma, K., Otsuka, M., Yamada, G., and Takahashi, H. (2017) Serum surfactant protein D predicts the outcome of patients with idiopathic pulmonary fibrosis treated with pirfenidone. *Respiratory medicine* **131**, 184-191
35. Greene, K. E., King, T. E., Jr., Kuroki, Y., Bucher-Bartelson, B., Hunninghake, G. W., Newman, L. S., Nagae, H., and Mason, R. J. (2002) Serum surfactant proteins-A and -D as biomarkers in idiopathic pulmonary fibrosis. *Eur Respir J* **19**, 439-446
36. Kodama, N., Yamaguchi, E., Hizawa, N., Furuya, K., Kojima, J., Oguri, M., Takahashi, T., and Kawakami, Y. (1998) Expression of RANTES by bronchoalveolar lavage cells in nonsmoking patients with interstitial lung diseases. *Am J Respir Cell Mol Biol* **18**, 526-531
37. Pardo, A., Gibson, K., Cisneros, J., Richards, T. J., Yang, Y., Becerril, C., Yousem, S., Herrera, I., Ruiz, V., Selman, M., and Kaminski, N. (2005) Up-regulation and profibrotic role of osteopontin in human idiopathic pulmonary fibrosis. *PLoS Med* **2**, e251
38. Kathiriya, J. J., Nakra, N., Nixon, J., Patel, P. S., Vaghasiya, V., Alhassani, A., Tian, Z., Allen-Gipson, D., and Dave, V. (2017) Galectin-1 inhibition attenuates profibrotic signaling in hypoxia-induced pulmonary fibrosis. *Cell Death Discov* **3**, 17010
39. Bennett, D., Bargagli, E., Bianchi, N., Landi, C., Fossi, A., Fui, A., Sestini, P., Refini, R. M., and Rottoli, P. (2020) Elevated level of Galectin-1 in bronchoalveolar lavage of patients with idiopathic pulmonary fibrosis. *Respir Physiol Neurobiol* **273**, 103323
40. Okamoto, M., Izuhara, K., Ohta, S., Ono, J., and Hoshino, T. (2019) Ability of Periostin as a New Biomarker of Idiopathic Pulmonary Fibrosis. *Adv Exp Med Biol* **1132**, 79-87

41. Yoshihara, T., Nanri, Y., Nunomura, S., Yamaguchi, Y., Feghali-Bostwick, C., Ajito, K., Murakami, S., Mawatari, M., and Izuhara, K. (2020) Periostin plays a critical role in the cell cycle in lung fibroblasts. *Respir Res* **21**, 38
42. Okamoto, M., Hoshino, T., Kitasato, Y., Sakazaki, Y., Kawayama, T., Fujimoto, K., Ohshima, K., Shiraishi, H., Uchida, M., Ono, J., Ohta, S., Kato, S., Izuhara, K., and Aizawa, H. (2011) Periostin, a matrix protein, is a novel biomarker for idiopathic interstitial pneumonias. *Eur Respir J* **37**, 1119-1127
43. Gorka-Dynysiewicz, J., Pazgan-Simon, M., and Zuwala-Jagiello, J. (2019) Pentraxin 3 Detects Clinically Significant Fibrosis in Patients with Chronic Viral Hepatitis C. *BioMed research international*
44. Diamond, J. M., Lederer, D. J., Kawut, S. M., Lee, J., Ahya, V. N., Bellamy, S., Palmer, S. M., Lama, V. N., Bhorade, S., Crespo, M., Demissie, E., Sonett, J., Wille, K., Orens, J., Shah, P. D., Weinacker, A., Weill, D., Kohl, B. A., Deutschman, C. C., Arcasoy, S., Shah, A. S., Belperio, J. A., Wilkes, D., Reynolds, J. M., Ware, L. B., Christie, J. D., and Lung Transplant Outcomes, G. (2011) Elevated plasma long pentraxin-3 levels and primary graft dysfunction after lung transplantation for idiopathic pulmonary fibrosis. *Am J Transplant* **11**, 2517-2522
45. Smith, D. R., Kunkel, S. L., Standiford, T. J., Rolfe, M. W., Lynch, J. P., 3rd, Arenberg, D. A., Wilke, C. A., Burdick, M. D., Martinez, F. J., Hampton, J. N., and et al. (1995) Increased interleukin-1 receptor antagonist in idiopathic pulmonary fibrosis. A compartmental analysis. *Am J Respir Crit Care Med* **151**, 1965-1973
