# Supplementary material for: Target inhibition of galectin-3 by inhaled TD139 in patients with idiopathic pulmonary fibrosis
Source: Eur Respir J. 2021 May 27;57(5):2002559. doi: 10.1183/13993003.02559-2020 (PMC8156151; doi:10.1183/13993003.02559-2020)
Supplement: Supplementary file 2 [file ERJ-02559-2020.FIGURES.pdf]

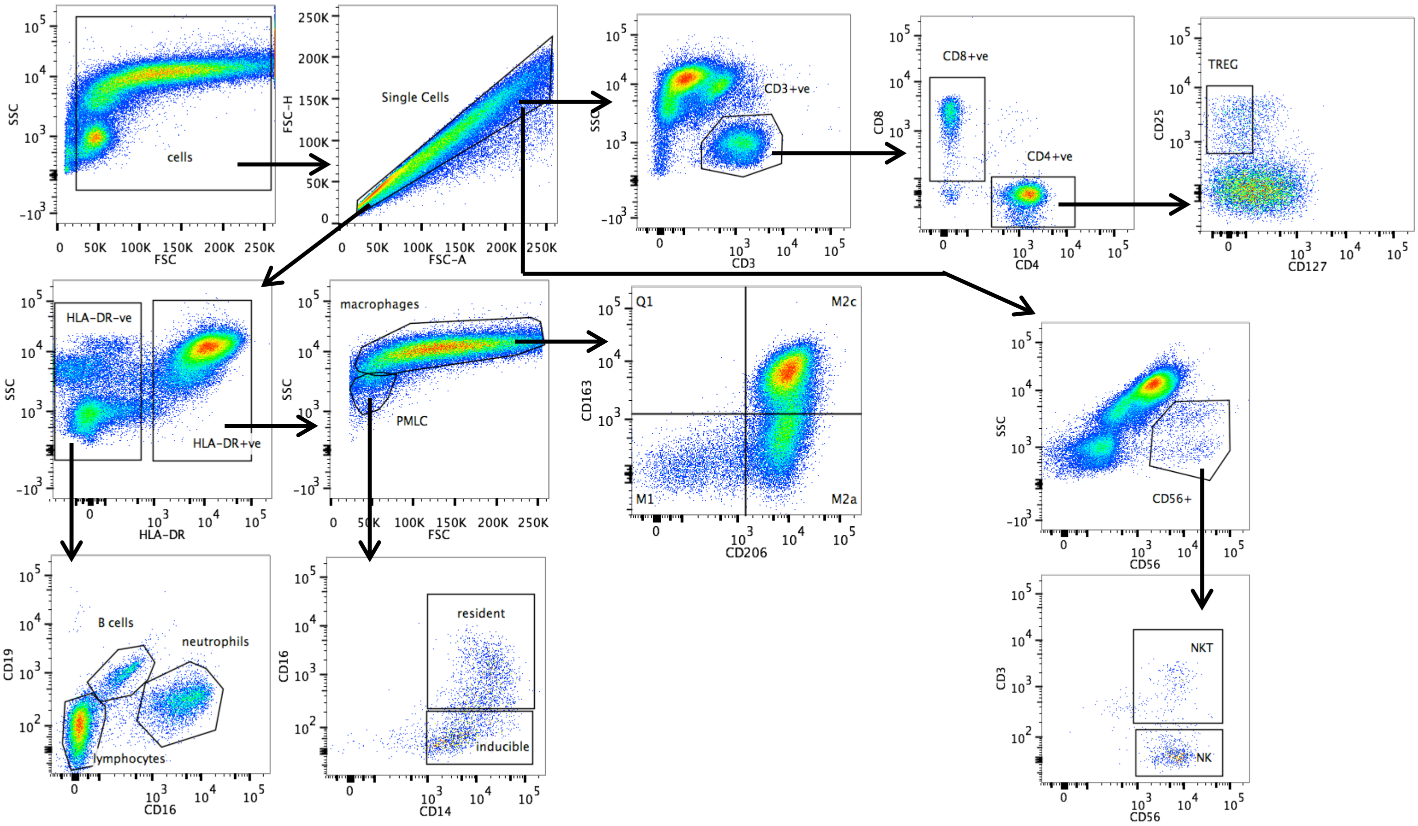

Figure S1. Flow cytometry analysis of BAL cells: gating strategy. After exclusion of doublets and debris, a sequential gating strategy was then employed to first identify populations expressing specific markers: $CD3^+/CD4^+$ and $CD3^+/CD8^+$ T cells. Tregs ($CD3^+/CD4^+/CD25^+/CD147^-$). HLA-$DR^+$ cells were subdivided into alveolar macrophages (high side scatter $CD163^+/CD206^+$ (M_{2c}), $CD163_{lo}/CD206^+$ (M_{2a}) and $CD163_{lo}/CD206_{lo}$ (M_1)) and monocytes (HLA-DR^+ low side scatter) were classified as resident ($CD14^+/CD16^+$) and recruited ($CD14^+/CD16^-$). Neutrophils (HLA-$DR^-/CD16^+$), B cells (HLA-$DR^-/CD19^+$), NK ($CD56^+/CD3^-$), NKT cells ($CD56^+/CD3^+$).

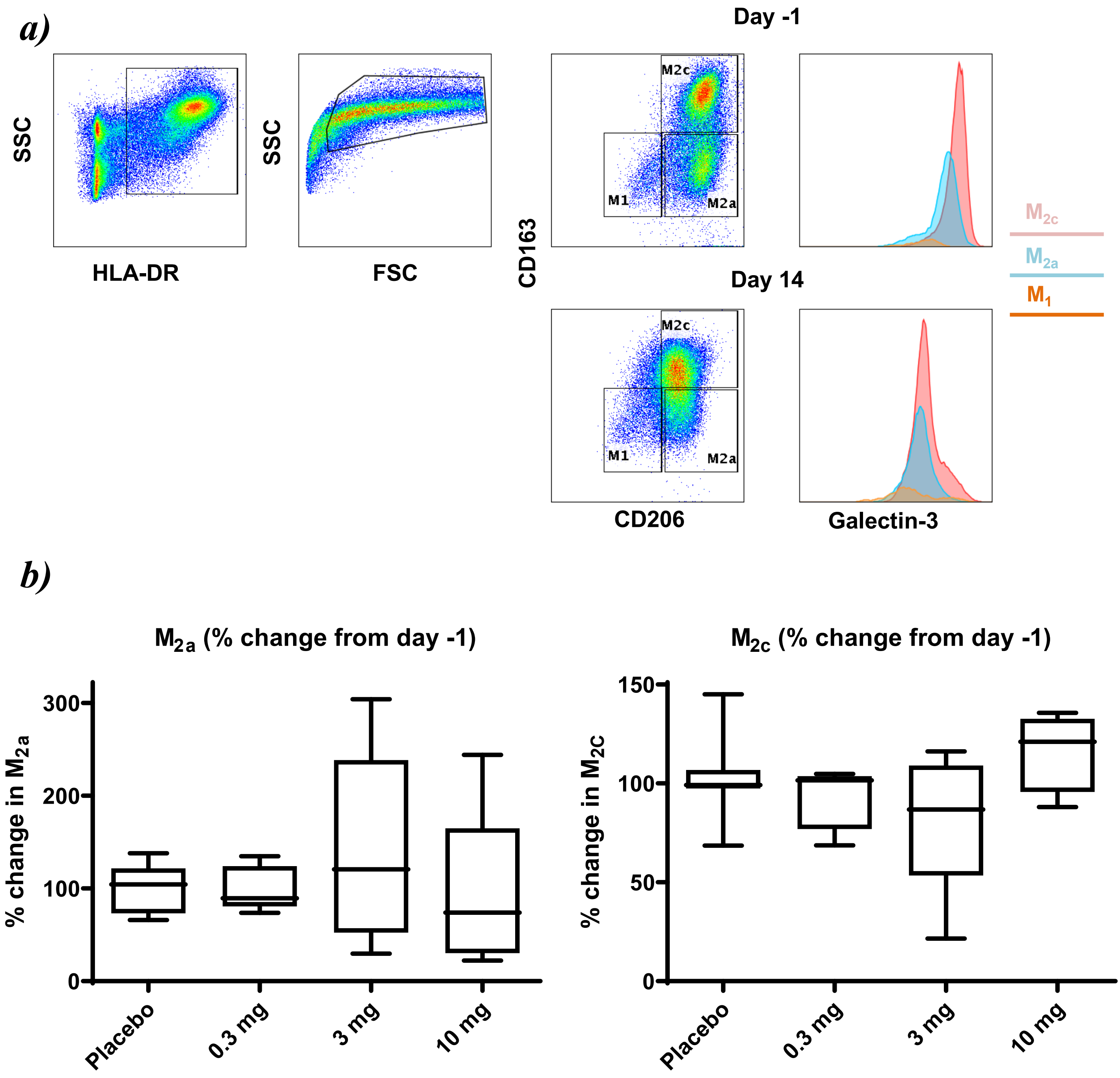

Figure S2. Flow cytometry analysis of BAL cells: Alveolar macrophage gating strategy. a) Representative alveolar macrophage gating strategy in a single patient pre-dose at day-1 and at day 14 at 2 h post-dose following 10 mg TD139 administration for 14 days. HLA-DR^{+} macrophages were classified as M_1, M_{2a} or M_{2c} based on CD206 and CD163 expression. Histograms show Galectin-3 expression on the different subsets M_{2a} pink, M_{2c} blue and M_1 orange on day -1 and day 14. b) % change in prevalence of M_{2a} and M_{2c} subsets from day -1 to day 14 in the different dose groups.

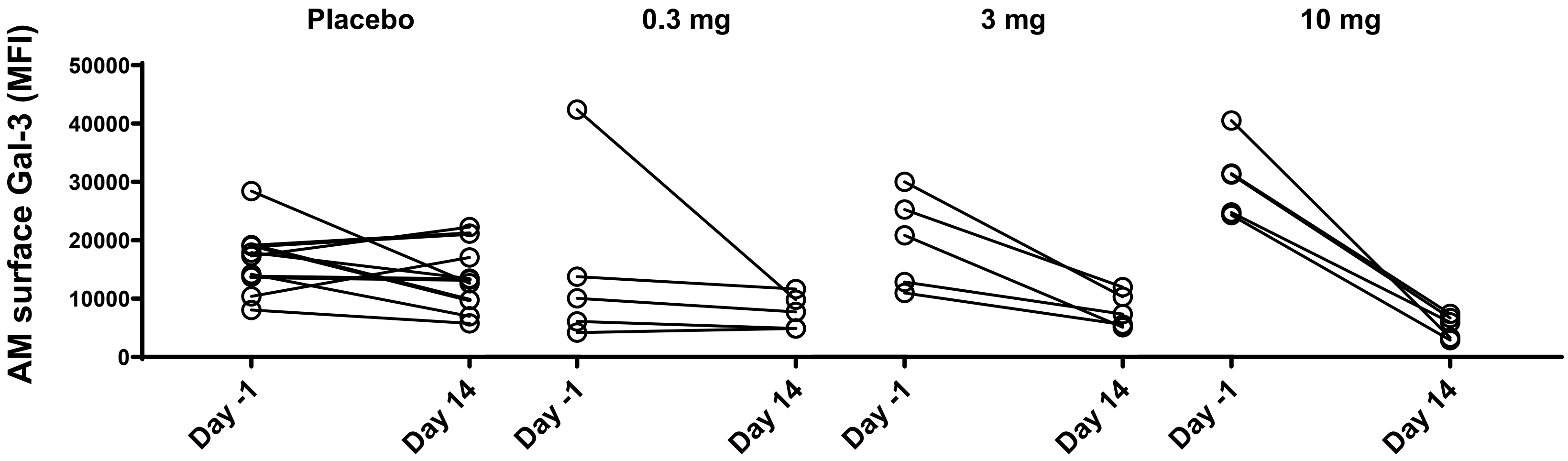

Figure S3. Galectin-3 changes in alveolar macrophages. Absolute change in surface macrophage galectin-3 on day -1 pre-dose or at 2 h post-administration of placebo, 0.3 mg, 3 mg and 10 mg of TD139 on day 14. AM, alveolar macrophages; MFI, mean fluorescence intensity.

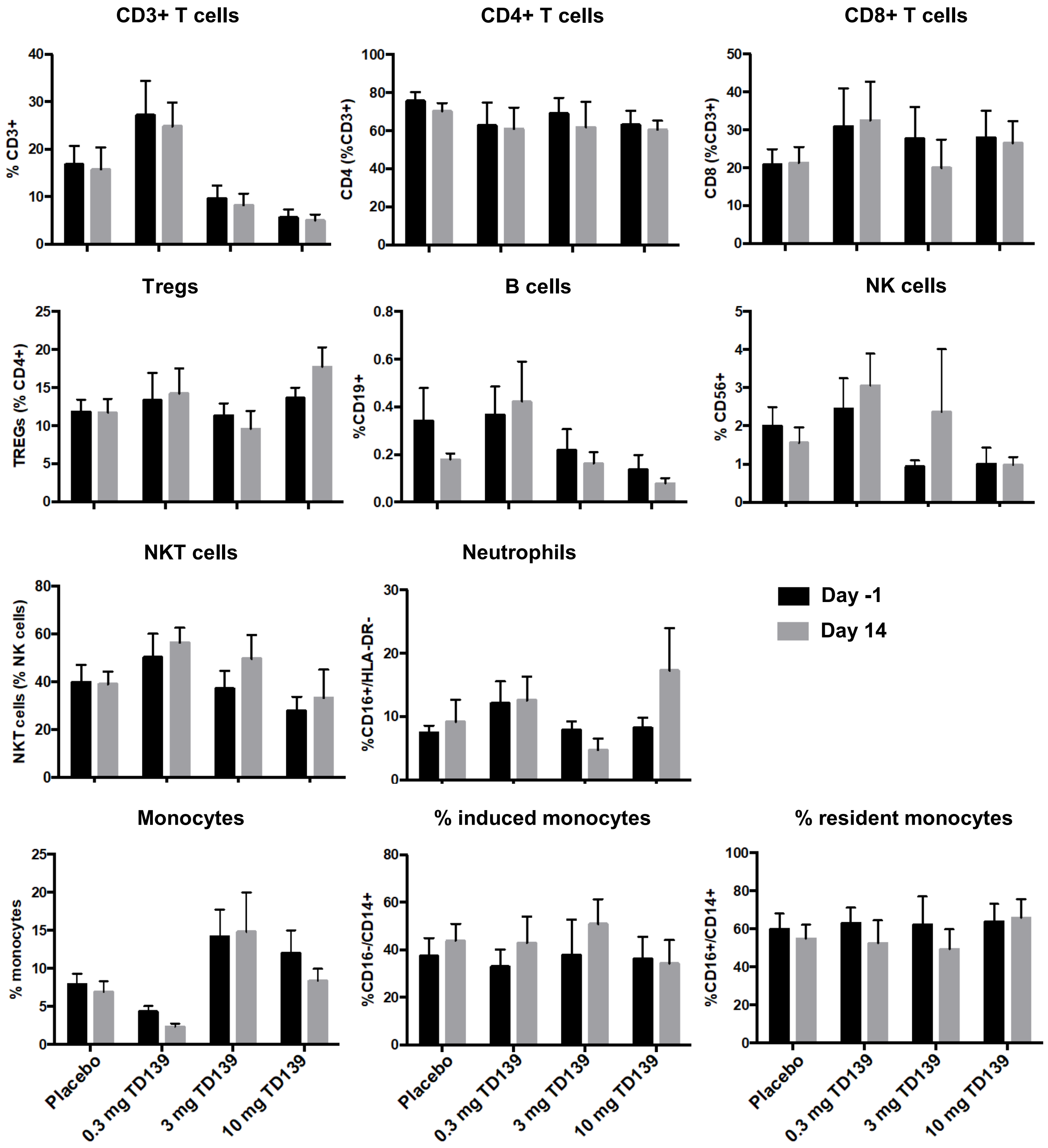

Figure S4. Flow cytometric analysis of immune cells in BAL. Cell types in BAL were identified as described in supplemental methods and outlined in figure S1. Results represent mean ± SEM from day -1 and day 14 samples from placebo and 0.3 mg, 3 mg and 10 mg TD139 groups.
