# Supplementary material for: Target inhibition of galectin-3 by inhaled TD139 in patients with idiopathic pulmonary fibrosis
Source: Eur Respir J. 2021 May 27;57(5):2002559. doi: 10.1183/13993003.02559-2020 (PMC8156151; doi:10.1183/13993003.02559-2020)
Supplement: Supplementary file 3 [file ERJ-02559-2020.Shareable.pdf]

SHAREABLE PDF

# Target inhibition of galectin-3 by inhaled TD139 in patients with idiopathic pulmonary fibrosis

Nikhil Hirani[1], Alison C. MacKinnon[1,2], Lisa Nicol[1], Paul Ford[2], Hans Schambye[2], Anders Pedersen[2], Ulf J. Nilsson[3], Hakon Leffler[4], Tariq Sethi[2], Susan Tantawi[2], Lise Gravelle[2], Robert J. Slack[2], Ross Mills[1], Utsa Karmakar[1], Duncan Humphries[1], Fredrik Zetterberg[2], Lucy Keeling[5], Lyn Paul[6], Philip L. Molyneaux[6], Feng Li[1], Wendy Funston[7], Ian A. Forrest[8], A. John Simpson[7,8], Michael A. Gibbons[9] and Toby M. Maher[6,10]

**Affiliations**: [1]MRC Centre for Inflammation Research, The Queen's Medical Research Institute, University of Edinburgh, Edinburgh, UK. [2]Galecto, Copenhagen, Denmark. [3]Dept of Chemistry, Lund University, Lund, Sweden. [4]Dept of Laboratory Medicine, Lund University, Lund, Sweden. [5]Exploristics, Belfast, UK. [6]National Institute for Health Research Respiratory Clinical Research Facility, Royal Brompton and Harefield NHS Foundation Trust, and Fibrosis Research Group, National Heart and Lung Institute, Imperial College London, London, UK. [7]Translational and Clinical Research Institute, Newcastle University, Newcastle upon Tyne, UK. [8]Respiratory Medicine Unit, Royal Victoria Infirmary, Newcastle upon Tyne Hospitals NHS Foundation Trust, Newcastle upon Tyne, UK. [9]Respiratory Dept, Institute of Biomedical and Clinical Science, Royal Devon and Exeter NHS Foundation Trust, Medical School, University of Exeter, Exeter, UK. [10]Keck School of Medicine, University of Southern California, Los Angeles, CA, USA.

**Correspondence**: Nikhil Hirani, MRC Centre for Inflammation Research, The Queen's Medical Research Institute, University of Edinburgh, 47 Little France Crescent, Edinburgh, EH16 4TJ, UK. E-mail: n.hirani@ed.ac.uk

@ERSpublications
**TD139 is a potent inhibitor of galectin-3, a key driver of fibrosis in the lung. In this phase 1/2a clinical study, inhaled TD139 was safe, well tolerated, and demonstrated target engagement and decreased plasma biomarkers associated with IPF progression.** https://bit.ly/2JREKx6

**Cite this article as:** Hirani N, MacKinnon AC, Nicol L, *et al*. Target inhibition of galectin-3 by inhaled TD139 in patients with idiopathic pulmonary fibrosis. *Eur Respir J* 2021; 57: 2002559 [https://doi.org/10.1183/13993003.02559-2020].

This single-page version can be shared freely online.

ABSTRACT Galectin (Gal)-3 is a profibrotic β-galactoside-binding lectin that plays a key role in the pathogenesis of idiopathic pulmonary fibrosis (IPF) and IPF exacerbations. TD139 is a novel and potent small-molecule inhibitor of Gal-3.

A randomised, double-blind, multicentre, placebo-controlled, phase 1/2a study was conducted to assess the safety, tolerability, pharmacokinetics and pharmacodynamics of inhaled TD139 in 36 healthy subjects and 24 patients with IPF. Six dose cohorts of six healthy subjects were evaluated (4:2 TD139:placebo ratio) with single doses of TD139 (0.15–50 mg) and three dose cohorts of eight patients with IPF (5:3 TD139: placebo ratio) with once-daily doses of TD139 (0.3–10 mg) for 14 days.

Inhaled TD139 was well tolerated with no significant treatment-related side-effects. TD139 was rapidly absorbed, with mean time taken to reach maximum plasma concentration ($C_{max}$) values ranging from 0.6 to 3 h and a plasma half-life ($T_{1/2}$) of 8 h. The concentration of TD139 in the lung was >567-fold higher than in the blood, with systemic exposure predicting exposure in the target compartment. Gal-3 expression on alveolar macrophages was reduced in the 3 and 10 mg dose groups compared with placebo, with a concentration-dependent inhibition demonstrated. Inhibition of Gal-3 expression in the lung was associated with reductions in plasma biomarkers centrally relevant to IPF pathobiology (platelet-derived growth factor-BB, plasminogen activator inhibitor-1, Gal-3, CCL18 and YKL-40).

Copyright ©ERS 2021. This version is distributed under the terms of the Creative Commons Attribution Licence 4.0.

TD139 is safe and well tolerated in healthy subjects and IPF patients. It was shown to suppress Gal-3 expression on bronchoalveolar lavage macrophages and, in a concerted fashion, decrease plasma biomarkers associated with IPF progression.
